# Heme regulates protein interactions and phosphorylation of BACH2 intrinsically disordered region in humoral response

**DOI:** 10.1016/j.isci.2024.111529

**Published:** 2024-12-04

**Authors:** Miki Watanabe-Matsui, Shun Kadoya, Kei Segawa, Hiroki Shima, Tadashi Nakagawa, Yuko Nagasawa, Shuichiro Hayashi, Mitsuyo Matsumoto, Mariko Ikeda, Akihiko Muto, Kyoko Ochiai, Long C. Nguyen, Katsumi Doh-Ura, Mikako Shirouzu, Keiko Nakayama, Kazutaka Murayama, Kazuhiko Igarashi

**Affiliations:** 1 Department of Biochemistry, Tohoku University Graduate School of Medicine, Sendai, Japan; 2 The Japan Society for the Promotion of Science (JSPS), Tokyo, Japan; 3 Pharmaceutical Discovery Research Laboratories, Teijin Pharma Limited, Tokyo, Japan; 4 Division of Cell Proliferation, ART, Tohoku University Graduate School of Medicine, Sendai, Japan; 5 Department of Clinical Pharmacology, Sanyo-Onoda City University, Sanyo-Onoda, Japan; 6 Laboratory for Protein Functional and Structural Biology, RIKEN Center for Biosystems Dynamics Research, Yokohama, Japan; 7 Department of Neurochemistry, Tohoku University Graduate School of Medicine, Sendai, Japan; 8 Division of Biomedical Measurements and Diagnostics, Tohoku University Graduate School of Biomedical Engineering, Sendai, Japan

**Keywords:** Biochemistry, Structural biology

## Abstract

Heme is known to bind to the intrinsically disordered region (IDR) to regulate protein function. The binding of heme to the IDR of transcription factor BACH2 promotes plasma cell differentiation, but the molecular basis is unknown. Heme was found to increase BACH2 IDR interaction with TANK-binding kinase 1 (TBK1). TBK1 inactivated BACH2 by phosphorylation of its IDR, whereas BACH2 repressed TBK1 gene expression. BACH2 phosphorylation by TBK1 inhibited its interaction with the co-repressor NCOR1 and promoted plasma cell differentiation. Heme also induced BACH2 binding to ubiquitin E3 ligase adaptor FBXO22, which polyubiquitinated BACH2 only in the presence of heme *in vitro*. Mutations of some of the TBK1-mediated phosphorylation sites promoted BACH2-FBXO22 interaction, while additional mutations abrogated their interaction, suggesting that TBK1 can both inhibit and promote BACH2-FBXO22 interaction. Therefore, heme regulates phosphorylation of BACH2 IDR by TBK1 and its interaction with NCOR1 and FBXO22, leading to de-repression of BACH2 target genes in humoral immunity.

## Highlights

• Heme enhances the direct interaction of BACH2 IDR with TBK1 and phosphorylation
• TBK1 inhibits BACH2 IDR-corepressors interaction depending on its kinase activity
• TBK1 and BACH2 form a double-negative feedback loop in plasma cell differentiation
• Heme promotes ubiquitination and degradation of BACH2 by FBXO22

## Graphical abstract

**Figure:**
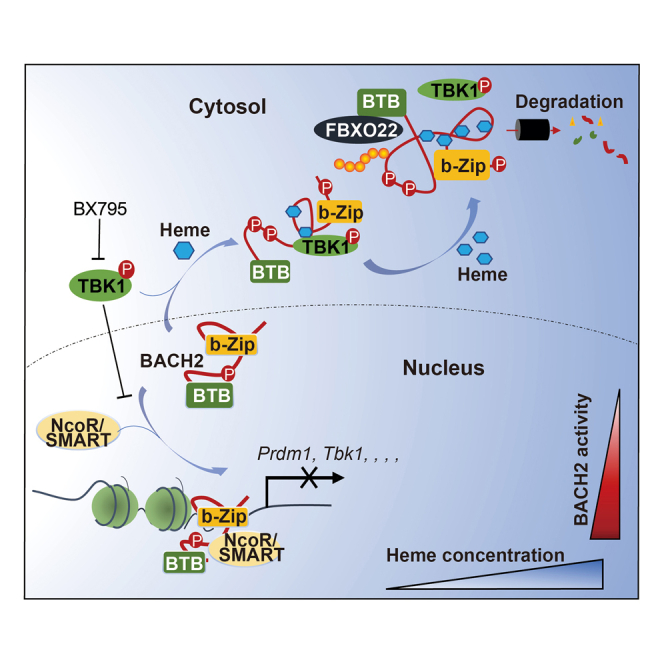

## Introduction

It is predicted that about 40% of all human proteins are intrinsically disordered proteins (IDPs) with at least one intrinsically disordered region (IDR), and about 25% are likely to have most of their regions in a disordered protein state.^1^^,^^2^ These widespread IDRs are thought to mediate interactions with other proteins to produce multifaceted functions.^3^ IDRs often serve as multi-specific binding sites that accommodate different binding partners,^4^ which can provide flexible and sensitive platforms for signal propagation and integration. Human transcription factors contain a high percentage of IDR (about 50% of their sequences), whereas prokaryotic transcription factors contain a low percentage of IDRs (about 5%–6%).^5^ This difference is thought to reflect the important functional roles played by IDRs in eukaryotes, including interaction with co-activators and the specificity of DNA binding.^6^^,^^7^ The combination of high specificity and low affinity of IDR-mediated protein interactions is thought to have many advantages, including increased responsibility to external environmental stimuli via post-translational modifications such as phosphorylation, increased flexibility to allow dynamic changes in the overall protein structure,^8^^,^^9^ and the capability to serve as a hub for multiple interactions.^10^^,^^11^^,^^12^

Heme is a crucial molecule in various biological processes.^13^^,^^14^^,^^15^^,^^16^^,^^17^ Heme proteins such as hemoglobin, myoglobin, and heme enzymes are characterized by a stable binding of heme to the well-structured regions of those proteins at a 1:1 stoichiometric ratio.^18^^,^^19^ The primary role of the heme moiety in their functions includes oxygen transport, electron transfer, and activation of oxygen for chemical modification. In addition to these archetypal functions of heme, recent studies have shown that heme fulfills a regulatory function within cells by binding to regulatory proteins including protein kinases and transcription factors and thus it is referred to as regulatory heme.^20^^,^^21^^,^^22^^,^^23^^,^^24^ It has also been suggested that heme functions as one of the damage-signals to alter inflammatory and immune responses.^25^^,^^26^ The possibility that heme is a signal is suggested by reports that heme binds to IDRs and regulates protein function.^27^^,^^28^^,^^29^^,^^30^ However, only a few of the target molecules of regulatory heme have been shown to play a physiological role in the immune system. It is also unclear how changes in the conformational state of IDRs to which heme binds lead to the regulation of protein function.

The transcriptional repressors BTB and CNC homolog BACH2 and BACH1 are structurally and evolutionarily related to each other and are both targets of regulatory heme; these proteins possess a basic-leucine zipper domain for DNA binding and multiple cysteine-proline (CP) motifs for heme binding.^31^^,^^32^^,^^33^ BACH2 is a critical regulator of development and responses of both B and T lymphocytes.^34^ BACH2 is required for class switch recombination (CSR) and somatic hypermutation of antibody genes in B cells, suppression of precocious differentiation to plasma cells, and differentiation of memory B cells.^34^^,^^35^^,^^36^^,^^37^ It is also required for differentiation of regulatory T cells and restriction of hyperactivation of effector T cells.^34^^,^^38^^,^^39^^,^^40^^,^^41^^,^^42^ A syndrome of BACH2-related immunodeficiency and autoimmunity (BRIDA) that results from *BACH2* haploinsufficiency has been reported,^43^ pointing to the clinical relevance of BACH2-mediated gene regulation. One of the important target genes of BACH2 in B and T cells is *Prdm1* that encodes the Blimp-1 transcription factor and promotes plasma cell differentiation in activated B cells and cytotoxic T cell development in T cells.^44^^,^^45^

In B cells, BACH2 restricts plasm cell differentiation by repressing the expression of *Prdm1*,^36^^,^^46^ whereas heme promotes their differentiation to plasma cells by inactivating BACH2 and thus inducing *Prdm1*.^32^ Unlike conventional heme proteins, heme-binding of BACH2 is mediated by its IDR.^32^^,^^47^^,^^48^ However, it is still unclear how heme binding to the BACH2 IDR alters its function as a transcription repressor. Several possible mechanisms can be envisioned for the regulation of BACH2 by the IDR-heme binding. The binding of heme to the BACH2 IDR may alter a protein interaction mediated by the IDR. For example, considering that the BACH2 IDR is rich in serine and threonine residues (22.6% in 331–520 amino acid [aa] region^47^), heme-binding may alter phosphorylation of the IDR and thus its protein interaction. Alternatively, heme binding to the IDR may affect a protein interaction outside the IDR.

In this study, we attempted to elucidate the mechanism of IDR regulation by heme using BACH2 as a model. We tried to examine the aforementioned possibilities by focusing on proteins that bind to the IDR or full-length BACH2 in the presence of heme. We combined purification of epitope-tagged BACH2 IDR from a B cell line followed by mass spectrometry to identify interacting proteins and *in vitro* binding assays using purified recombinant proteins to examine the effect of heme upon their interactions. We further used the stable isotope labeling using amino acids in cell culture (SILAC) to profile the entire interactants of full-length BACH2 and their responses to heme. The results suggest that heme regulates multiple protein interactions mediated by IDR or a region outside of IDR to regulate plasma cell differentiation.

## Results

### Heme-dependent interaction of BACH2 IDR with TBK1

**Figure 1:**
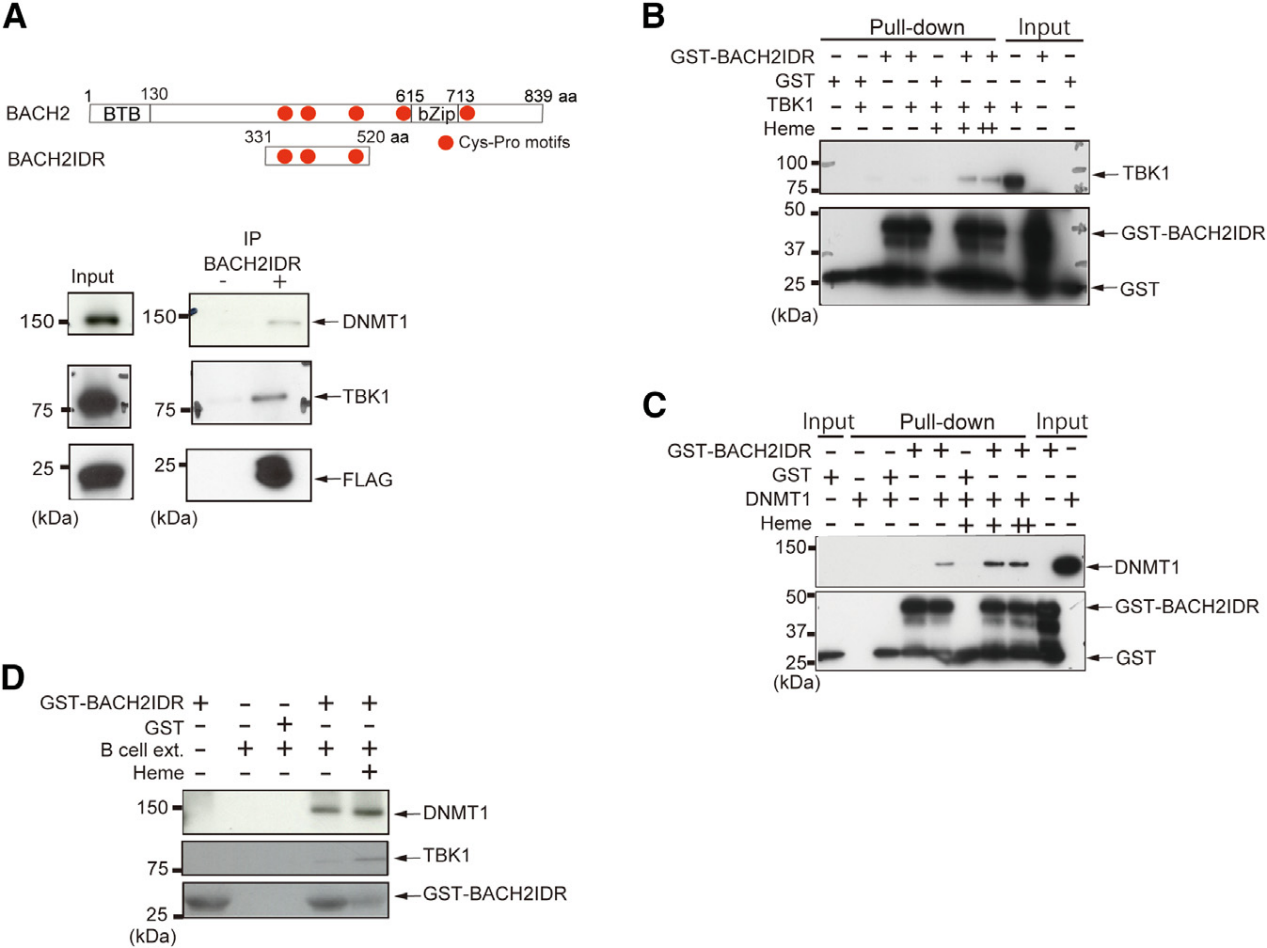
Interactions of BACH2 IDR with TBK1 and DNMT1 (A) Upper: a schematic representation of mouse BACH2 and BACH2-IDR. Lower: the immunoblot analysis of the affinity-purified samples using an anti-FLAG, anti-TBK1 or anti-DNMT1 antibody. (B) The *in vitro* pull-down assay using recombinant GST, GST-BACH2-IDR, or His-FLAG-TBK1 in the presence or absence of 5 μM or10 μM heme. GST and GST-BACH2-IDR were revealed with an anti-GST antibody. TBK1 was revealed with an anti-TBK1 antibody. (C) The *in vitro* pull-down assay using recombinant GST, GST-BACH2-IDR, or His-FLAG-DNMT1 in the presence or absence of 5 μM or 10 μM heme. GST and GST-BACH2-IDR were revealed with an anti-GST antibody. DNMT1 was revealed with an anti-DNMT1antibody. (D) The *in vitro* pull-down assay using recombinant GST, GST-BACH2-IDR, and B cell extracts in the presence or absence of 10 μM heme. GST and GST-BACH2-IDR were revealed with anti-GST an antibody. TBK1 was revealed with an anti-TBK1 antibody. DNMT1 was revealed with an anti-DNMT1 antibody.

The IDR of BACH2 accepts multiple heme molecules, altering its conformational state without inducing any secondary structure.^47^^,^^48^ Even without the induction of secondary structure, such a structural change may affect the protein binding capacity of the IDR or other regions of BACH2. To identify proteins that bind to the BACH2 IDR and mediate the effects of heme upon BACH2, we performed a purification of proteins interacting with FLAG-HA-tagged BACH2 IDR: the region of amino acid residues 331–520 of BACH2 (Figure 1A). FLAG-HA-BACH2-IDR was stably expressed in BAL17 mature B cells using a retrovirus expression system and was purified along with its interacting proteins by using the ReCLIP method^49^ and an antibody against the FLAG epitope. The proteins co-purified with FLAG-HA-BACH2-IDR were identified by liquid chromatography-tandem mass spectrometry (LC-MS/MS). We focused on proteins that were identified in the FLAG-HA-BACH2-IDR sample but were absent from the control purification from cells infected with the virus without FLAG-HA-BACH2-IDR. The list of these proteins included TBK1, KDM1, HUWE1, DNMT1, and other proteins (Table 1). There were significant differences (*p* < 0.05) in the numbers of peptide matches of these proteins between the purifications with and without FLAG-HA-BACH2-IDR. The presence of TBK1 and DNMT1 in the FLAG-HA-BACH2-IDR complex was verified by immunoblotting analyses using TBK1 and DNMT1-specific antibodies (Figure 1A). To confirm their direct binding and to examine the possible effect of heme on their interaction, we performed an *in vitro* pull-down assay using the recombinant GST-BACH2-IDR, FLAG-His-TBK1 and FLAG-His-DNMT1 proteins in the presence or absence of heme. We found that GST-BACH2-IDR directly interacted with TBK1 and DNMT1 and their interactions were enhanced in the presence of heme (Figures 1B and 1C). The interaction of BACH2 with TBK1 or DNMT1 was also increased by heme when BAL17 extracts were examined as a source of TBK1 and DNMT1 (Figure 1D). FLAG-HA-BACH2-IDR contains three CP motifs, suggesting that these heme-binding motifs may be involved in the regulation of the interaction of BACH2 and TBK1 by heme. However, a smaller fragment of BACH2 spanning 381–481 aa and lacking these three CP motifs still bound to TBK1 in a heme-dependent manner (Figure S1). These results indicated that the BACH2 IDR interacts with TBK1 in a heme-dependent and CP motif-independent manner. Since the BACH2 IDR contains many phosphorylation sites and TBK1 is a serine/threonine kinase of the IKK family and is an important factor for the innate immune response and the activation of nuclear factor κB (NF-κB),^50^^,^^51^^,^^52^ we hypothesized that TBK1 might regulate BACH2 in the acquired immune response.

**Table 1:** The list of proteins identified as BACH2_IDR interacting proteins using ReCLIP

*Gene name*	Protein	Protein hit no.	Protein family member	Molecular mass	Protein score	Spectral count (unique)	No. of sequence
*BACH2*	Transcription regulator protein BACH2	49	1	80,968	1,536	54	9
*TBK1*	Serine-threonine-protein kinase TBK1	624	1	84,153	133	3	2
*KDM1*	Lysine-specific histone demethylase 1A	609	1	93,432	139	4	4
*UBR4*	E3 ubiquitin-protein ligase UBR4	677	1	580,593	474	20	13
*HUWE1*	E3 ubiquitin-protein ligase HUWE1	204	1	487,234	464	14	9
*DNMT1*	DNA (cytosine-5)-methyltransferase 1	363	1	185,986	246	14	11
*BRG1*	Transcription activator BRG1	207	1	181,953	448	22	9
*USP19*	Ubiquitin carboxyl-terminal hydrolase 19	450	1	152,656	193	9	4
*USP7*	Ubiquitin carboxyl-terminal hydrolase 7	532	1	129,743	161	8	6
*MBD3*	Methyl-CpG-binding domain protein 3	562	1	32,432	151	3	2
*SMARCA5*	SWI/SNF-related matrix-associated actin-dependent regulator of chromatin subfamily A member 5	143	1	122,474	645	29	22
*CPSF1*	Cleavage and polyadenylation specificity factor subunit 1	145	1	162,350	643	23	9
*CNOT1*	CCR4-NOT transcription complex subunit 1	225	1	269,549	417	18	6
*MAP1S*	Microtubule-associated protein 1S	241	1	104,154	389	10	8
*ARID1A*	AT-rich interactive domain-containing protein 1A	360	1	243,076	247	6	3
*IDH3B*	isocitrate dehydrogenase 3 (NAD+) beta	361	1	42,523	247	3	1
*SBNO2*	Protein strawberry notch homolog 2	397	1	150,763	225	2	3
*GTF2I*	General transcription factor II-I	434	1	112,834	202	13	6
*POLR1A*	DNA-directed RNA polymerase I subunit RPA1	461	1	196,544	188	8	4
*ILF2*	Interleukin enhancer-binding factor 2	473	1	43,319	184	7	5
*BTAF1*	TATA-binding protein-associated factor 172	521	1	209,619	164	4	3
*FAM120A*	Constitutive coactivator of PPAR-gamma-like protein 1	591	1	123,061	143	4	4
*PPP4R1*	Serine/threonine-protein phosphatase 4 regulatory subunit 1	596	1	108,290	143	3	2
*CSTF1*	Cleavage stimulation factor subunit 1	592	1	49,345	143	5	5
*KIF4*	Chromosome-associated kinesin KIF4	491	1	141,422	175	7	3

The proteins that were identified in the FLAG-HA-BACH2 IDR ReCLIP experiment with a protein score of >130 but were not detected in the mock experiment are listed in order of protein hit number, irrespective of their protein scores. Protein hit number indicates the rank of the MASCOT protein family (a group of proteins sharing at least one peptide) to which each protein belonged. Protein family member indicates the protein score rank of each protein in the protein family to which it belonged. Protein score indicates the reliability of the protein identification derived from sum of the high set ions score for each sequence match that exceeds the threshold where ions score is −10 logP, and P is the probability that the MS/MS spectral match is a ramdo event.

### BACH2-TBK1 double-negative feedback loop in B cells

**Figure 2:**
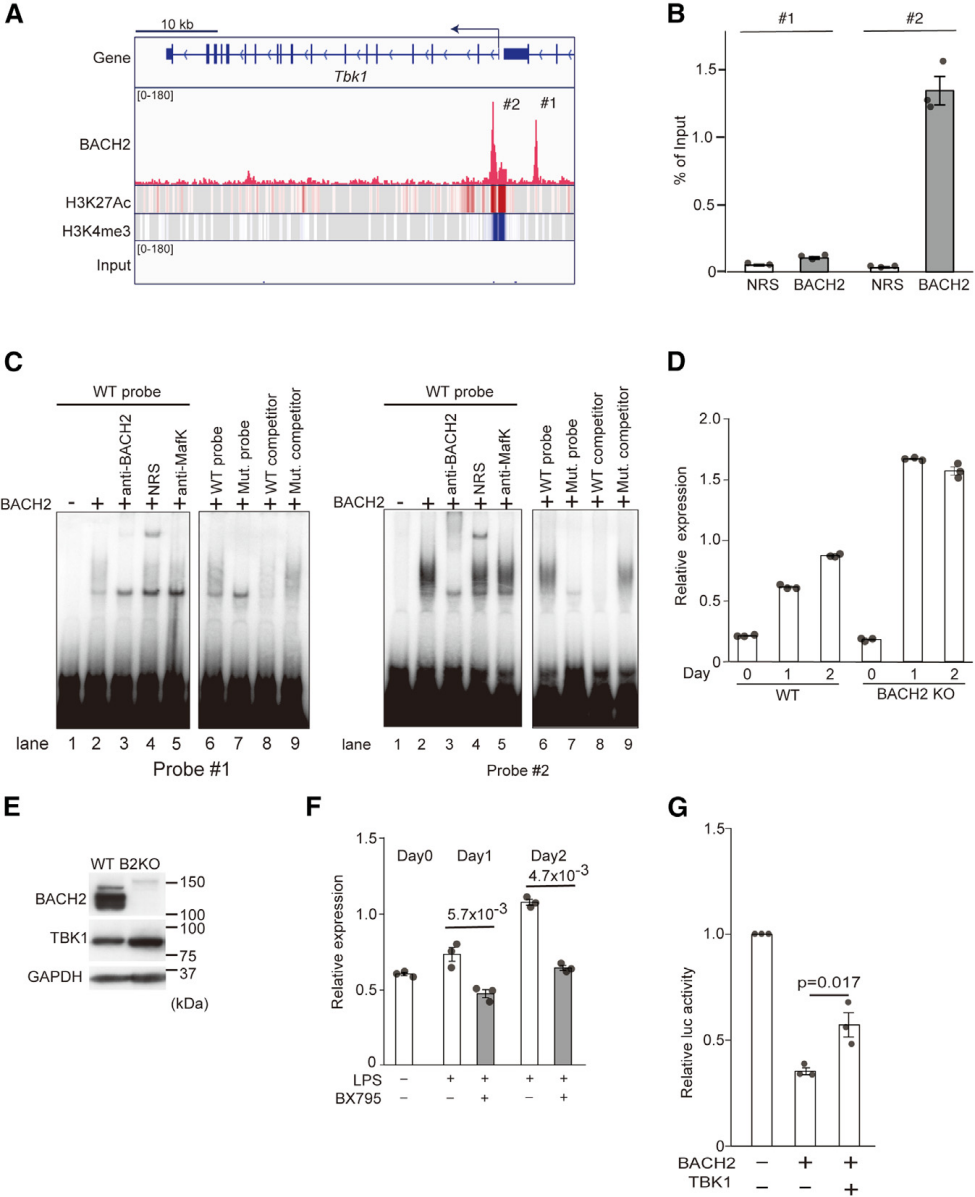
Identification of *Tbk1* gene as a direct target of BACH2 (A) The ChIP-seq binding profiles for BACH2 in Ebf1 KO pre-pro-B cell line (GSE87503) and those of histone H3 lysine 27 acetylation (H3K27Ac) and histone H3 lysine 4 trimethylation (H3K4me3) in mouse primary B cell (GSM1441280 and GSM1441328 in GSE60103). The ChIP-seq data were visualized using Integrative Genomics Viewer (IGV v2.11.1^55^). Two BACH2-binding sites were found in the promoter (#1) and intron (#2) regions of the *Tbk1* gene. Blue arrow indicates the transcription start site (TSS) of the *Tbk1* gene. (B) The binding of BACH2 to sites #1 and #2 was demonstrated by ChIP-qPCR in the BAL17 mature B cell line. The results are shown as enrichment relative to samples immunoprecipitated by NRS. (C) The electrophoretic mobility shift assay showing the binding of BACH2 to the MERE-like sequences found in BACH2-binding sites #1 (left) and #2 (right). The ^32^P-labeled probes were mixed with the BACH2 and MAFK proteins without any additive (lane 2) or in the presence of an anti-BACH2 antibody (lane 3, anti-BACH2), a normal rabbit serum (lane 4, NRS) or an anti-MafK antiserum (lane 5, anti-MafK). Competition assays using the unlabeled wild-type or mutant probes as competitors were also shown (lanes 6 and 7, no competitor, lane 8, wild-type competitor and lane 9, Mut competitor). The results shown here are representative of those from three experiments. (D) The RT-qPCR analysis of the TBK1 mRNA expression in wild-type and BACH2 KO mouse splenic B cell stimulated with LPS (20 μg/mL) for 1 or 2 days. (E) The western blotting results of wild-type and BACH2 KO mouse splenic B cell. BACH2 was revealed with an anti-BACH2 antibody. TBK1 was revealed with an anti-TBK1 antibody. GAPDH served as an internal control. (F) The effect of BX795 on the TBK1 mRNA expression in wild-type and BACH2 KO splenic B cell stimulated with LPS (20 μg/mL) in the presence or absence of 1 μM BX795 for 1 or 2 days. (G) HEK293T cells were transfected with the indicated reporter and effector plasmids. The amounts of plasmids were as follows: pGL4-4.28- *Slc48a1*MARE luciferase reporter (2.0 μg), pEF seapansy (10 ng), pCMV BACH2 (200 ng), and pcDNA FLAG 3.1 TBK1 (200 ng).

To find clues to the significance of the BACH2-TBK1 interaction, firstly we used the data from our previously reported chromatin immunoprecipitation sequencing (ChIP-seq) analysis of BACH2 in B cells.^53^ By revisiting those data, we found two peaks of BACH2 binding within the *Tbk1* gene itself, one of which was located at the promoter upstream region and the other downstream of the transcription start site (the #1 and #2 sites, respectively; Figure 2A). The #2 site was accompanied by histone H3 lysine 27 acetylation (H3K27Ac) and H3K4 tri-methylation (H3K4Me3) that are involved in gene expression controls.^54^ Quantitative PCR-based ChIP assays with an anti-BACH2 antibody revealed that BACH2 binding was more prominent at the #2 site than the #1 site in BAL17 cells (Figure 2B). In electrophoretic mobility shift assay (EMSA), recombinant BACH2 clearly bound to the #2 site, whereas its binding to the #1 site was substantially weaker compared with the #2 site (Figure 2C). The binding to the #2 site was abolished with a BACH2 antibody and the unlabeled wild-type probes but not with the ones with mutations, confirming the specificity of the BACH2 binding.

To examine whether the *Tbk1* gene is repressed by BACH2 in B cells, we isolated splenic B cells from wild-type and *Bach2*-deficient (KO) mice and stimulated the cells with lipopolysaccharide (LPS) *in vitro*. While the *Tbk1* mRNA was induced upon LPS stimulation of wild-type B cells, its induction was higher in *Bach2* KO B cells than wild-type B cells (Figure 2D). Consistent with this observation, the TBK1 protein was expressed in a larger amount in *Bach2* KO splenic B cells than in wild-type B cells (Figure 2E). The induction of the *Tbk1* mRNA was found dependent upon the TBK1 kinase activity inasmuch as a treatment with a TBK1 inhibitor BX795 resulted in a significant downregulation of *Tbk1* gene expression in wild-typesplenic B cells (Figure 2F). These results suggest that the *Tbk1* gene is directly repressed by BACH2 and is upregulated upon B cell activation through phosphorylation of BACH2 by TBK1 itself.

Considering that TBK1 interacted with BACH2 in a heme-dependent manner, we next examined whether TBK1 would regulate genes involved in heme metabolism. As a model target gene of BACH2 in this context, we focused on the *Slc48a1* gene encoding the heme transporter HRG1, which facilitates transport of heme from endolysosomal compartments to cytoplasmic region.^56^^,^^57^ BACH2 was found to bind to the *Slc48a1* MARE in ChIP analysis (Figure S2A). In EMSA, recombinant BACH2 clearly bound to the *Slc48a1* MARE (Figure S2B). Furthermore, the *Slc48a1* mRNA was increased in *Bach2*-deficient B cells (Figure S2C). Therefore, *Slc48a1* is a direct target gene of BACH2. The *Slc48a1* reporter carrying the *Slc48a1* MARE sequence was repressed by expression of BACH2 and/or MAFK in 293T cells, which was relieved by the co-expression of TBK1 (Figures 2G and S2D). These results suggested that TBK1 inhibits the repressor activity of BACH2 in B cells. Therefore, TBK1 and BACH2 form a double-negative feedback loop in which TBK1 and BACH2 sustain their activities by repressing each other’s activity, leading to either stable repression or expression of BACH2 target genes such as *Slc48a1*.

### Promotion of plasma cell differentiation by TBK1

**Figure 3:**
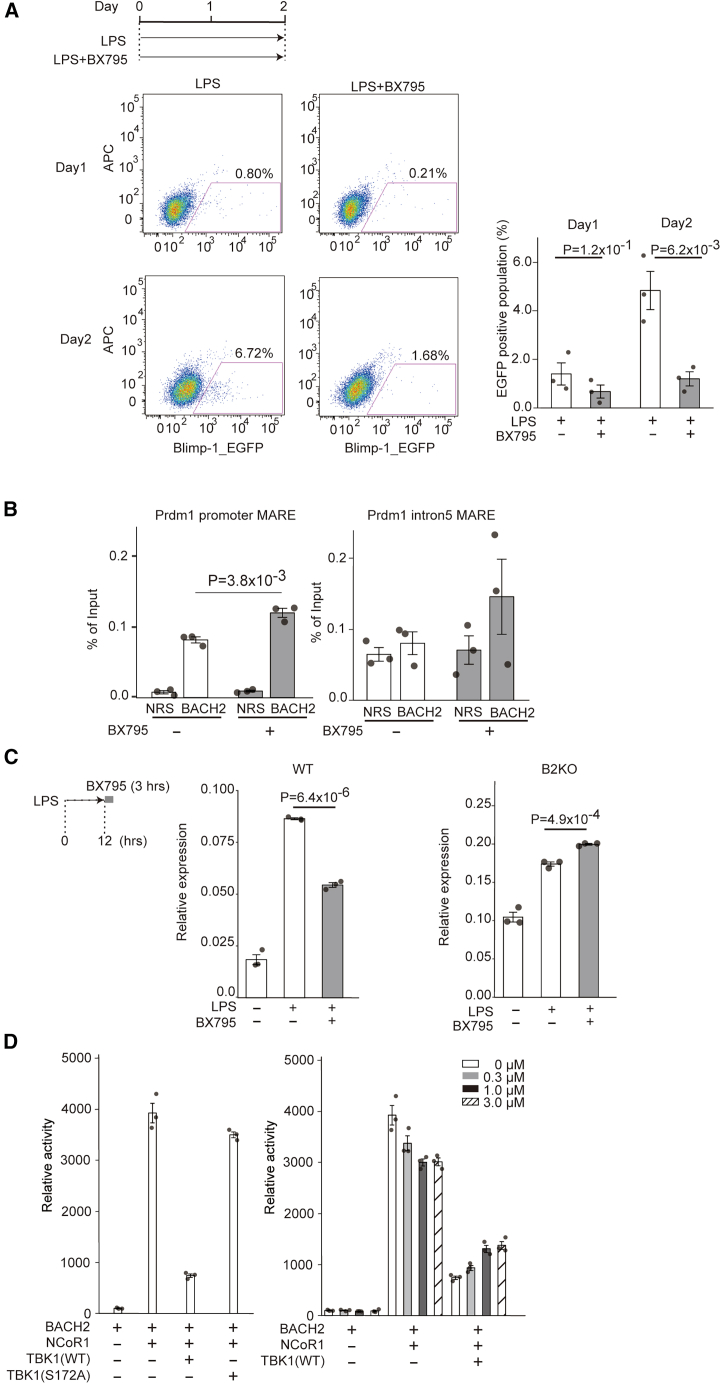
TBK1 promotes plasma cell differentiation by inactivating BACH2 (A) Upper left: a schematic representation of the experimental design. Lower left and right panels: BX795 repressed the *Blimp-1* gene expression in mouse splenic B cells. Lower left: The expression of the *Blimp-1*-EGFP reporter gene determined by a FACS analysis. B220-positive cells from the wild-type *Blimp-1*-EGFP mice were stimulated with 20 μg/mL LPS in the presence or absence of 1 μM BX795 on day 1 or day 2. Each gate shows the percentage of EGFP-positive cells. Representative results of a triplication are shown in right panel. The experiment was performed with *N* = 3. Right: the percentage of EGFP-positive cells cultured with LPS+BX795 or LPS alone in wild-type B cells. The data are presented as the means ± SD of triplicate determinations. The statistical analyses were performed by the use of the Student’s t test. The experiment was performed with *N* = 3. (B) The binding of BACH2 to the *Prdm1* promoter (left panel) and *Prdm1* intron5 (right panel) regions was demonstrated by ChIP-qPCR in the BAL17 mature B cell line. BAL17 cells were treated with BX795 for 3 h. Results are shown as enrichment relative to sample immunoprecipitated by a normal rabbit serum (NRS). The experiment was performed with *N* = 3. (C) The precursor mRNA expression of *Prdm1* in wild-type and BACH2 KO splenic B cells treated with BX795 for 3 h was quantified by RT-qPCR. After the stimulation by LPS, the cells were cultured for 12 h before the treatment. The quantities of *Prdm1* were normalized to the β-actin mRNA. The data are mean ± SD of three independent experiments. The experiment was performed with *N* = 3. (D) The mammalian two-hybrid (M2H) assay showing the inhibitory effect of TBK1 and TBK1-S172A on the interaction of BACH2 with NCoR1 (left). The inhibition of the interaction by TBK1 was diminished by a TBK1 inhibitor (right). HEK293T cells were co-transfected with the pGL4.35-9×GAL4UAS-Luc reporter (Promega) vector, pFN26A-hBACH2 GAL4-DBD fusion expression vector and pFN10A-hNCOR1 VP-16 AD expression vector together with either pCAG-hTBK1 or pCAG hTBK1(S172A). After 24 h of incubation, the cells were treated with the TBK1 inhibitor MRT67307 at the indicated concentrations of 0–3.0 μM for 6 h. After that, the cells were lysed, and luciferase activity was measured. The results are shown as fold induction compared to the negative control (pGL4.35-9×GAL4UAS-Luc alone) and represent the mean of triplicates from a representative experiment, with error bars showing the standard deviation.

We next investigated whether TBK1 would regulate plasma cell differentiation of B cells by inactivating BACH2. BACH2 represses the *Prdm1* gene, which is a master regulator of plasma cell differentiation. We examined whether inhibition of TBK1 activity would alter *Prdm1* gene expression using a transgenic mouse with a reporter EGFP gene embedded in the *Prdm1* locus that strictly reflects plasma cell differentiation.^36^^,^^58^ We isolated splenic B220-positive B cells from these mice, stimulated them with LPS for 1 or 2 days to activate and to induce plasma cell differentiation, and determined the percentage of the EGFP-positive cell population by using FACS. EGFP-positive cells appeared after 2 days, whereas additional treatment with a TBK1 inhibitor BX795 almost abolished the differentiation of the EGFP-positive plasma cells (Figure 3A). These results suggested that TBK1 was required for *Prdm1* expression in activated B cells and hence plasma cell differentiation.

To examine whether TBK1 induced the expression of *Prdm1* by inactivating BACH2 in B cells, we carried out ChIP analysis of BACH2 using BAL17 cells in the presence or absence of BX795. The amounts of BACH2 bound at the promoter MARE of the *Prdm1* gene were higher in the presence of BX795 than in the absence of BX795 (Figure 3B). BX795 tended to increase BACH2 binding to the intron MARE of *Prdm1*, but the difference was not statistically significant (Figure 3B). To investigate whether TBK1 would hinder the repression of *Prdm1* by BACH2, we compared responses of splenic B cells isolated from wild-type and BACH2 KO mice to BX795 (Figure 3C). Firstly, the splenic B cells were stimulated with LPS for 12 h and then were treated with BX795 for another 3 h. To assess the effect of the drug on the ongoing transcription, the newly synthesized *Prdm1* mRNA precursor was measured using quantitative RT-PCR analysis with primers to amplify one of its exon-intron junctions. While the treatment with LPS clearly increased the amount of the precursor mRNA, BX795 treatment resulted in its significant reduction in wild-type B cells. In contrast, there was no difference in the amounts of the precursor mRNA in BACH2 KO B cells in the presence or absence of BX795 (Figure 3C). Taken together, these results suggested that TBK1 promoted plasma cell differentiation by negating the BACH2-mediated repression of the *Prdm1* gene.

### Disruption of BACH2-corepressor interaction by TBK1

We hypothesized that the BACH2-TBK1 interaction may affect the interaction of BACH2 with NcoR1, which acts as a BACH2 co-repressor.^59^ We addressed this possibility using a mammalian two-hybrid (M2H) assay with 293T cells. BACH2 interacted with NCOR1 (Figure 3D, left). This interaction was abrogated by the expression of wild-type TBK1 but not by a TBK1(S172A) mutant that lost the kinase activity.^60^ The TBK1 inhibitor MRT67307 partially reversed the effect of TBK1 upon its interaction (Figure 3D, right). Therefore, TBK1 reduced the BACH2-NCOR1 interaction depending on its kinase activity. We also found that the interaction of BACH2 with NCOR2 (also known as SMRT), another co-repressor closely related to NCOR1, was also reduced by TBK1 (Figure S3). These results suggest that the phosphorylation of BACH2 by TBK1 inhibits its interaction with co-repressors and hence repression of BACH2 target genes.

### Identification of FBXO22 as E3 ligase of heme-bound BACH2

**Figure 4:**
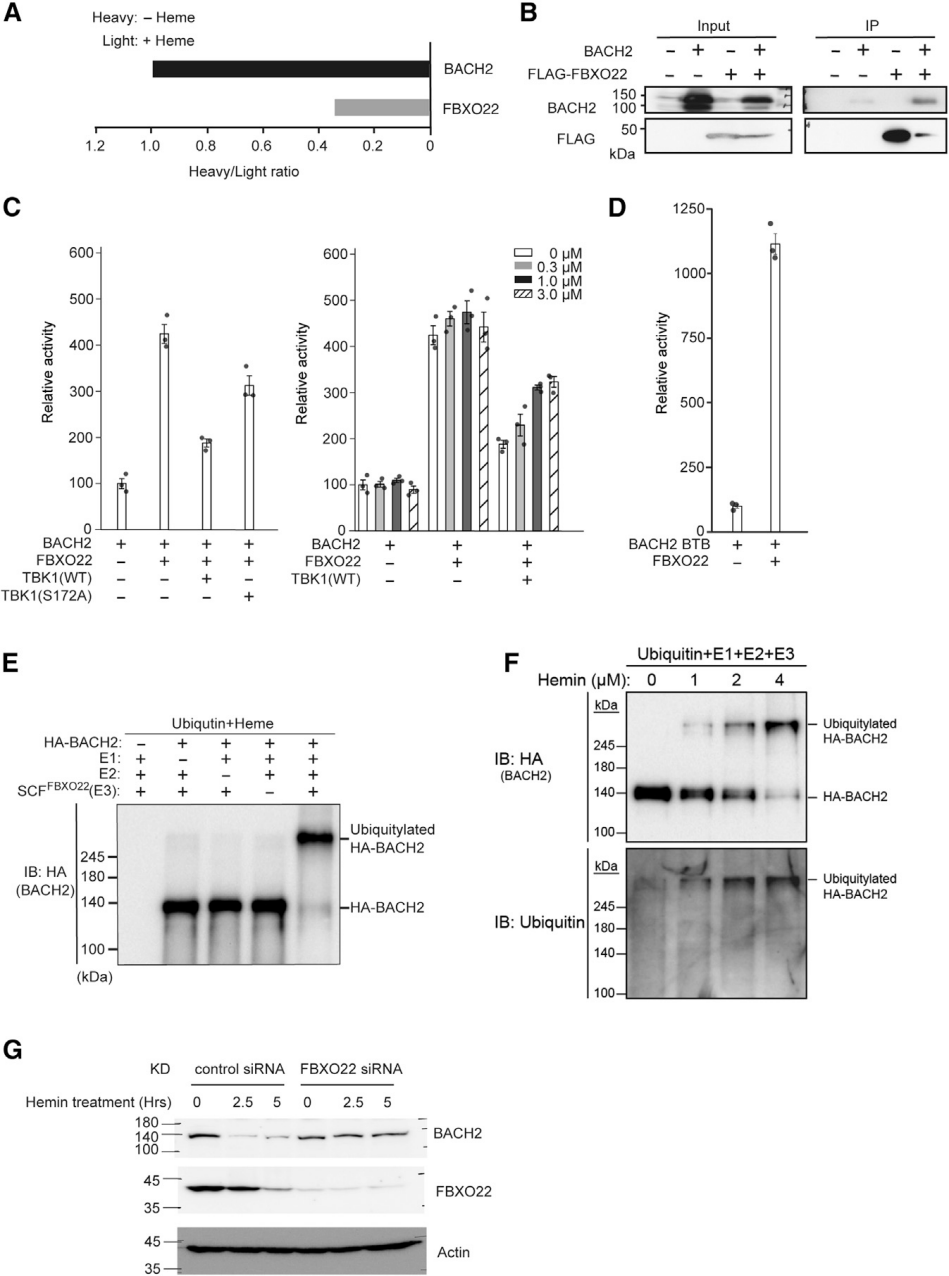
Heme-dependent interaction of BACH2 with FBXO22 (A) A comparison of the Heavy/Light ratio of BACH2 and FBXO22 in the SILAC experiment. (B) The interaction of mouse FBXO22 with BACH2. FLAG-FBXO22 and BACH2 were overexpressed in HEK293T cells. FLAG-FBXO22 was immunoprecipitated with an anti-FLAG antibody. The resulting samples were analyzed by immunoblotting using anti-FLAG or anti-BACH2 antibodies. (C) The M2H assay with HEK293T cells in the presence or absence of the TBK1 inhibitor MRT67307 at 0 to 3.0 μM. HEK293T cells were co-transfected with the indicated combinations of the pFN26A-hBACH2 GAL4 DBD fusion expression vector, pFN10A-hFBXO22 VP-16 AD fusion expression vector, pGL4.35-9×GAL4UAS-Luc reporter vector (Promega), pCAG-hTBK1 and pCAG-hTBK1(S172A). After 24 h of incubation, the cells were treated with the TBK1 inhibitor MRT67307 at 0 to 3.0 μM for 6 h. After that, the cells were lysed, and luciferase activity was measured. The results are shown as fold induction compared to the negative control (pGL4.35-9×GAL4UAS-Luc alone) and represent the mean of triplicates from a representative experiment, with error bars showing the standard deviation. (D) The M2H assay showing the interaction of the BTB domain of BACH2 with FBXO22. pFN26A-hBACH2 BTB and pFN10A-hFBXO22 plasmids were transfected into HEK293T cells. After that, each sample was used for the M2H assay. The results are shown as in (C). (E and F) The western blotting analysis indicating that the SCF^FBXO22^ E3 ligase complex promotes HA-BACH2 polyubiquitination in a heme-dependent manner. Anti-HA or anti-ubiquitin antibodies were used. (G) Namalwa cells were transfected with control or FBXO22 siRNAs and were treated with hemin for the indicated periods in the presence of cycloheximide. Proteins were detected using the indicated antibodies.

Heme also induces BACH2 degradation in B cells.^32^ To clarify the mechanism involved, we tried to identify ubiquitin ligases or their adopter proteins that bound to BACH2 in response to heme. Considering that such proteins may bind to a region outside of the BACH2 IDR, we expressed full-length BACH2 with a FLAG tag in BAL17 cells for an affinity purification of BACH2. We compared BACH2-interacting proteins in BAL17 cells with or without heme in the culture medium using SILAC-based quantitative LC-MS/MS to determine the relative quantity of peptides labeled with stable isotopes (H) and unlabeled (L) peptides (Tables S1 and S2). Several FBXO E3 ligase adaptor proteins including FBXO22^61^^,^^62^ were found to interact with BACH2. In recent reports, FBXO22 was found to mediate the heme-induced degradation of BACH1.^63^^,^^64^ The interaction of BACH2 with FBXO22 was increased in the presence of heme, suggesting the involvement of FBXO22 in BACH2 degradation (Figure 4A). Co-immunoprecipitation analysis of FLAG-FBXO22 and BACH2 overexpressed in 293T cells confirmed their interaction (Figure 4B).

Next, we examined the possible involvement of TBK1 in the interaction of BACH2 and FBXO22 using M2H assay in 293T cells. While BACH2 interacted with FBXO22, co-expression of wild-type TBK1 inhibited the BACH2-FBXO22 interaction (Figure 4C, left). In contrast, TBK1 (S172A) mutant showed a smaller effect on the BACH2-FBXO22 interaction than wild-type TBK1. The effect of TBK1 was reversed by the TBK1 inhibitor MRT67307 (Figure 4C, right). Consistent with its binding to the BTB domain of BACH1,^63^ FBXO22 was found to interact with the BTB domain of BACH2 (Figure 4D). These results indicated that BACH2 and FBXO22 interacted in B cells in a heme-regulated manner and that their interaction was reduced by TBK1-mediated phosphorylation of BACH2.

We next investigated whether FBXO22 functioned as an adaptor of ubiquitin E3 ligase toward BACH2 using an *in vitro* ubiquitination assay. In this experiment, we expressed full-length BACH2 tagged with an HA epitope in 293T cells and purified it to use as a substrate in a purified ubiquitination assay system. As shown in Figures 4E and 4F, a mobility-shift of HA-BACH2 to higher molecular weight species was detected only in the presence of heme. We confirmed that the shift was due to ubiquitination by using anti-ubiquitin antibodies (Figure 4F). Prior phosphorylation of HA-BACH2 by TBK1 did not affect the *in vitro* ubiquitination of HA-BACH2 by FBXO22 (Figure S4A). These results established that FBXO22 is an E3 ligase adaptor for ubiquitination of heme-bound BACH2. FBXO22 knockdown in mouse splenic B cells resulted in an increase of the BACH2 protein (Figure S4B). When Namalwa cells, derived from human B cell lymphoma cell, were treated with hemin in the presence of cycloheximide to block new synthesis of proteins, BACH2 protein was decreased. These reductions were suppressed when FBXO22 was knocked down with siRNA (Figure 4G). These results showed that FBXO22 regulated BACH2 protein amount in B cells as a ubiquitin E3 ligase adaptor in response to heme.

### Restriction of nuclear accumulation and protein amount of BACH2 by TBK1

**Figure 5:**
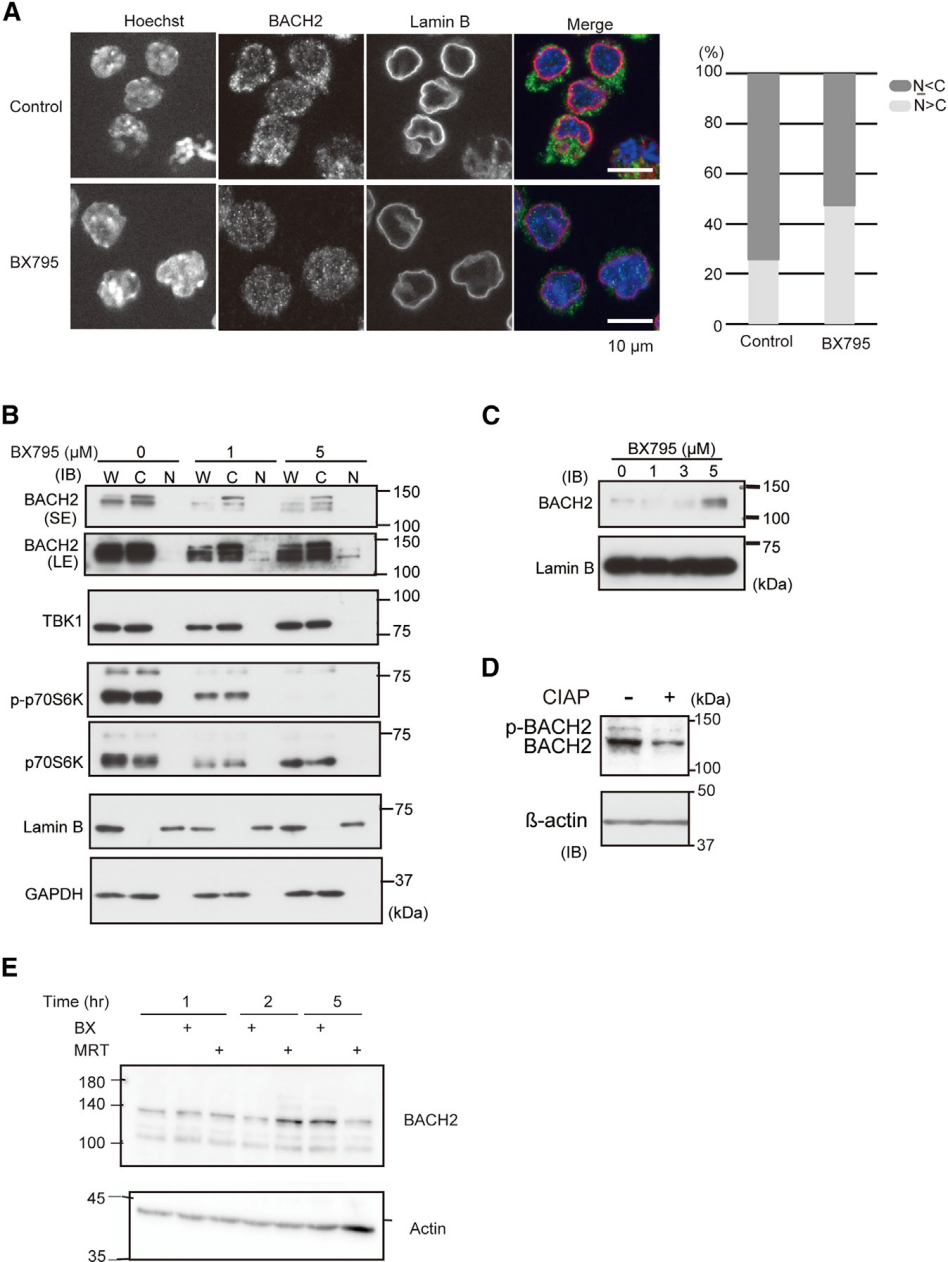
The localization of BACH2 is regulated by TBK1 (A) Immunocytochemistry for the BACH2 protein in the presence of BX795 in M1 cells. The subcellular localization of BACH2 was evaluated by classification of 120 cells for each condition into two classes: cytoplasm dominant (N < C) and nucleus dominant (N > C). Blue represents nuclei stained by Hoechst, green represents BACH2 and red represents lamin B1. Scale bar represents 10 μm. (B) The subcellular localization of BACH2 in mouse splenic B cells cultured in the presence of BX795. Cells were cultured in the presence of LPS (20 μg/mL) for 12 h and then were treated with 1 or 5 μM BX795 for 3 h. BACH2 was revealed with an anti-BACH2 antibody (SE, short exposer; LE, long exposer). TBK1 was revealed with an anti-TBK1 antibody. p70S6K and p-p70S6K were revealed with anti-p70S6K and anti-p-p70S6K antibodies. Lamin B1 served as a control for the nuclear fraction and GAPDH was used as a control for the cytoplasmic fraction. W, whole-cell extract; C, cytoplasm; N, nucleus. (C) The dose-dependent effect of BX795 on the nuclear accumulation of BACH2 in mouse splenic B cells. Cells were cultured in the presence of LPS (20 μg/mL) for 20 h and then were treated with 1, 3 or 5 μM BX795 for 3 h. BACH2 was revealed with an anti-BACH2 antibody and lamin B1 served as a control for the nuclear fraction. (D) The western blotting results of the BACH2 protein before and after a CIAP-treatment of the whole-cell extracts from M1 cells. β-actin served as an internal control. (E) Namalwa cells were treated with the TBK1 inhibitors (2 μM) for the indicated periods, and BACH2 and actin were detected by western blotting.

The subcellular localization of BACH2 is regulated by mammalian target of rapamycin (mTOR) and the protein kinases downstream of phosphatidylinositol-3 kinase (PI3K).^65^^,^^66^ This raised the question of whether TBK1 would also regulate the subcellular localization of BACH2. In order to examine this possibility, we compared the subcellular localization of endogenous BACH2 in the presence or absence of BX795 by immunofluorescence staining using M1 cells that express BACH2 abundantly. BACH2 was present mainly in the cytoplasmic region with an exclusion from the nuclear region in nearly 70% of the cells (Figure 5A). In contrast, the intense staining of BACH2 in the cytoplasmic region disappeared in the presence of BX795 and showed clear nuclear staining in roughly 50% of the cells. To confirm these observations, we prepared nuclear and cytoplasmic extracts from the splenic B cells that were treated *in vitro* with 1 or 5 μM BX795 for 12 h. Irrespective of the BX795 treatment, most of the BACH2 protein was present in the cytoplasmic fraction (Figure 5B). In the presence of BX795, a faster-migrating band of BACH2 appeared, which was also detected in the nuclear fraction (Figure 5B). Using only the nuclear extracts used in Figure 5B, we confirmed the accumulation of BACH2 in the nucleus when cells were treated with BX795 (Figure 5C). While BACH2 was present mostly in the cytoplasmic fraction upon the biochemical fractionation, this can be an overestimation due to leakage from nuclei during the procedure. A treatment of the extract with calf intestine alkaline phosphatase (CIAP) diminished the upper band of BACH2 (Figure 5D), indicating that the upper band contained phosphorylated BACH2.

We next examined whether TBK1 affected BACH2 protein amount. Namalwa cells were treated with two TBK1 inhibitors (BX795 or MRT67307), and endogenous BACH2 protein was examined (Figure 5E). Both inhibitors increased the amount of BACH2 protein. Since the effects were transient and the kinetics of BACH2 accumulation was different, a feedback regulation may be present in response to an accumulation of the BACH2 protein and/or TBK1 inhibition. These results suggested that TBK1 was involved in the phosphorylation and inactivation of BACH2 in B cells.

### Heme modulates phosphorylation sites of BACH2 IDR

**Figure 6:**
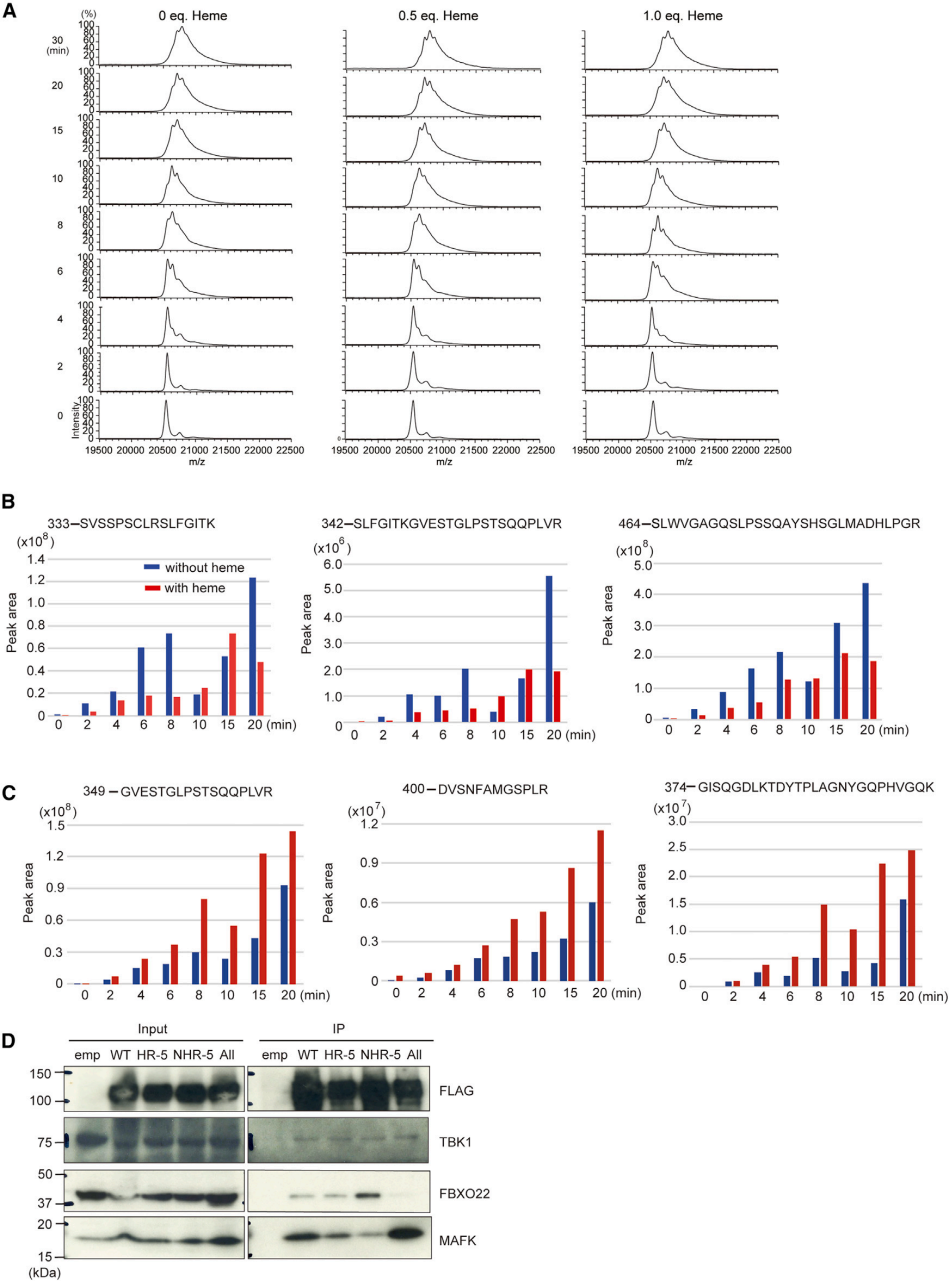
Binding of heme to BACH2 IDR regulates phosphorylation site-specificity of TBK1 (A) The mass shift of the BACH2 IDR fragment after the TBK1 kinase assay was examined by MALDI-TOF mass spectrometry. The reactions were done using 10 μM BACH2 IDR with TBK1 for different reaction times (0–30 min) in the presence or absence of heme (0, 5, and 10 μM). (B and C) Quantification of TBK1-mediated phosphorylation in BACH2 IDR in the presence or absence of heme. The BACH2 IDR protein after the TBK1 kinase assay was analyzed by LC-MS/MS. The sequences of six representative mono-phosphorylated peptides of BACH2 identified by a MASCOT search are shown with the *m*/*z* and *z* numbers of the precursor ions. The red characters in the peptide sequences stand for the most probable phosphorylation site of each peptide. Quantification of the precursor ions was done based on their extracted ion chromatograms obtained from the MS/MS data and is shown in the graphs. The blue and red bars indicate the absence and presence of heme in the assay condition, respectively. (D) HEK293T cells were transfected with the expression plasmids for FLAG-BACH2 (wild-type) and FLAG-BACH2 mutants (HR-5, NHR-5, and All) and FLAG-BACH2 was immunoprecipitated from the cell lysates with an anti-FLAG antibody coupled to magnetic beads. The immunoblot analyses were carried out with the indicated antibodies.

We investigated whether TBK1 directly phosphorylated BACH2 and, if so, how phosphorylation would be affected by heme. To compare the kinetics of phosphorylation with or without heme, we measured phosphorylation states of BACH2 IDR (after removal of GST) using an *in vitro* kinase assay with various incubation times in the presence or absence of heme followed by matrix assisted laser desorption-ionization-time of flight (MALDI-TOF) mass spectroscopy. Since we measured molecular mass without protein digestion into peptides, the stoichiometry of phosphorylation can also be quantitated.^67^ As shown in Figure 6A, mass shift was clearly detected in a time-dependent manner that reflected phosphorylation of BACH2 IDR by His-FLAG-TBK1. The shift patterns were consistent with phosphorylation at multiple sites, up to 3 or 4 phosphorylations per one BACH2 IDR molecule. However, the patterns of a mass shift and their transitions along the incubation time were similar in the presence or absence of heme. These results raised the possibility that heme might alter the specificity of phosphorylated residues rather than the overall amount of phosphorylation.

To address the aforementioned possibility, we next examined whether heme would affect the site-specificity of TBK1 using LC-MS/MS. In this analysis, each phosphorylation reaction was stopped with 0.5% TFA and was treated with DTT and acrylamide, and then was digested with trypsin. The phosphopeptides in the reaction samples were enriched by TiO2-affinity purification and were subjected to an LC-MS/MS analysis. This analysis identified multiple phosphorylation sites on BACH2. By monitoring phosphorylation over time, we found that phosphorylation at several sites was clearly altered by the presence or absence of heme (Figures 6B, 6C, and S5). TBK1 is known to phosphorylate amino acid sequences containing a Ser-Leu motif,^68^ which is also present in BACH2. Some of the Ser-Leu motifs present in BACH2 were phosphorylated more rapidly in the absence of heme than in its presence (Figure 6B). In the presence of heme, TBK1 tended to phosphorylate different Ser and Thr residues (Figure 6C). These results suggest that heme binding to the BACH2 IDR altered its structural state, leading to a modulation of substrate recognition by TBK1.

To determine whether the identified phosphorylation sites of BACH2 by TBK1 affect the protein-protein interaction of BACH2 with TBK1 or FBXO22, we generated three phospho-mutants of BACH2 (Figure S5). The first derivative FLAG-BACH2-NHR-5 contained alanine substitutions of Ser or Thr residues whose phosphorylation by TBK1 was not altered by heme (non-heme-regulated-5 sites [NHR-5]; S342A, T347A, S352A, S464A, and S472A). The second derivative FLAG-BACH2-HR-5 contained alanine substitutions of Ser and Thr that were efficiently phosphorylated by TBK1 in the presence of heme (heme-regulated-5 sites [HR-5]; S352A, S357A, T382A, S402A, and S472A). FLAG-BACH2-All contained combined mutations of eight sites of these two derivatives (S342A, T347A, S352A, S357A, T382A, S402A, S464A, and S472A). We transfected into HEK293T cells with the wild-type or the mutant plasmids and performed immunoprecipitation experiments with an anti-FLAG antibody. As shown in Figure 6D, the NHR-5 mutant showed more interaction with FBXO22 compared with the wild-type or HR-5. In contrast, the interaction of NHR-5 with TBK1 was not altered. The combination of these mutations abolished FBXO22 binding. These results suggest that TBK1-mediated phosphorylation of BACH2 IDR at NHR-5 restricts the BTB domain-mediated interaction with FBXO22, whereas further phosphorylation at HR-5 promotes their interaction. There may be different pathways for the state of phosphorylation of BACH2 by TBK1 in the presence or absence of heme.

## Discussion

Several studies have suggested that heme regulates the responses of acquired immunity.^32^^,^^69^^,^^70^^,^^71^ Such a regulatory heme may be leaked from cells within damaged tissues or may reflect an increased synthesis of heme upon immune cell activation.^25^ While BACH2 and its related factor BACH1 have been established as heme-regulated transcription factors, including the CP motif-mediated heme binding^32^^,^^72^ and structural changes in its IDR induced by heme binding,^32^^,^^47^^,^^73^^,^^74^ it has remained unclear how such structural changes would lead to the alterations in its functions as a transcription factor. A major obstacle to solving this problem has been that it is not clear how heme-binding affects the interaction of BACH2 with other proteins. In this study, we addressed this issue by using the ReCLIP method,^49^ which is expected to allow capturing a transient and dynamic protein-protein interaction. We have shown that binding of heme to the BACH2 IDR induces changes in protein-protein interactions and TBK1-mediated phosphorylation. This may lead to de-repression of its target genes and promotion of plasma cell differentiation.

Using pull-down assays of purified recombinant proteins or B cell extracts, we found that heme enhanced the physical interactions between BACH2 IDR and TBK1 or DNMT1 (Figure 1). However, because the purified BACH2 IDR was extremely unstable in solution and its structural state was not homogeneous, it turned out very difficult to determine its binding kinetics using biophysical methods such as surface plasmon resonance or isothermal titration calorimetry. Hence, the effect of heme on exact K_D_ values of the interactions between the BACH2 IDR and TBK1 or DNMT1 remains unclear. Nonetheless, our biochemical analysis clearly established direct, heme-stimulated interactions of the BACH2 IDR with these proteins. To determine the binding constants of BACH2 IDR to TBK1 or DNMT1, it will be necessary to identify the binding regions of these factors using peptides of various lengths, including the BACH2 IDR. Then, we may be able to determine the coupling constants to the BACH2 IDR by performing time course NMR measurements. While the BACH2 protein complex was examined previously, its interaction with TBK1 or DNMT1 was not detected.^59^^,^^66^ IDRs frequently engage in interactions with high specificity but low affinity (in the range of ∼μM), which allows rapid exchange of binding sites between multiple interacting partners.^12^ Since protein kinases are known to interact with their substrates only transiently,^75^ such interactions may elude conventional purification of protein complexes and may require methods like ReCLIP for identification, which we employed here. It should be noted that TBK1 also phosphorylates BACH1 within its IDR and promotes its degradation,^64^ even though BACH1 and BACH2 IDRs do not show sequence similarity. There may be a structural similarity of their IDRs that allow binding and recognition by TBK1. As we delved into the BACH2-TBK1 interaction in this study, the biological significance of the BACH2-DNMT1 interaction awaits further studies.

Beyond its essential function in the regulation of innate immune response,^50^^,^^76^ TBK1 is known to inhibit class-switch recombination in B cells.^77^ However, the downstream substrates of TBK1 in B cells have remained unclear. The present observations described here place BACH2 as a TBK1 substrate for CSR and plasma cell differentiation, with two critical interactions constituting a mutually inhibitory regulatory loop. First, BACH2 binds to the TBK1 promoter region and directly represses its transcription. Second, TBK1 directly phosphorylates BACH2 to inactivate its activity as a transcription repressor (discussed further in the following). These two regulatory interactions constitute a mutually repressing positive feedback loop, raising the possibility that BACH2 may be able to reduce its own phosphorylation by TBK1 via repressing TBK1 expression. As a corollary to this, TBK1 may be able to maintain its expression by overriding the BACH2-mediated repression of its gene. How is this regulatory network important in B cells? While BACH2 promotes germinal center (GC) reactions, including CSR of antibody genes, and restricts plasma cell differentiation in activated B cells by repressing the expression of *Prdm1*,^35^^,^^46^^,^^58^ it has been unclear how BACH2-mediated repression of *Prdm1* is released once activated B cells are ready for plasma cell differentiation. Recently, TBK1 has been found to play a role as a B cell-intrinsic factor for GC formation.^78^ TBK1 may promote GC reactions and then inactivate BACH2 to allow plasma cell differentiation. This possibility was supported by our present finding that the TBK1 inhibitor reduced *Prdm1* expression and thus suppressed plasma cell differentiation of B cells activated *in vitro*. Since BACH2 and *Prdm1* also form a mutually repressing positive feedback loop,^36^ the integration of the two regulatory loops (BACH2 and TBK1, and BACH2 and *Prdm1*) is expected to allow a toggle switch-like regulation of plasma cell differentiation, with a clear demarcation of B and plasma cell states in terms of gene expression. Since TBK1 also induced the expression of *Slc48a1* by inactivating BACH2, heme content is expected to increase by HRG1-mediated import in activated B cells. Once this cycle is initiated, the BACH2 repressor activity may be rapidly reduced, leading to the terminal differentiation to plasma cells.

We also discovered that heme promoted FBXO22-mediated polyubiquitination of BACH2 *in vitro*. While FBXO22 was shown to promote heme-induced degradation of BACH1 in lung cancer cells,^63^ the detailed mechanism of their interaction remains unclear. Our results using a purified reconstituted system clearly established that FBXO22 promotes polyubiquitination of heme-bound BACH2, which may also hold for BACH1. The two related findings are worth further discussion. First, FBXO22 was found to bind to the BTB domain of BACH2. While the BTB domain does not bind heme,^32^ BACH2-FBXO22 interaction was nonetheless increased by heme. Therefore, some structural changes induced by heme binding to BACH2 IDR may alter the positioning of the BTB domain within the overall structure of BACH2 molecule, leading to its recognition by FBXO22. Second, TBK1 reduced the interaction between BACH2 and FBXO22 depending on its catalytic activity in the two-hybrid assays. Phosphorylation of BACH2 IDR may make the BTB domain unrecognizable for FBXO22. While the results of the mutation analysis of BACH2 IDR support that phosphorylation of BACH2 regulates its interaction with FBXO22, more detailed analyses are required, because the multiple mutations may affect the structural state of the BACH2 IDR. Notwithstanding this limitation, our present observations strongly suggest that the overall spatial arrangement of BACH2 molecule may be regulated by phosphorylation and heme binding within BACH2 IDR.

Since the interaction between BACH2 and TBK1 was enhanced by the presence of heme *in vitro*, the key question was whether phosphorylation of BACH2 by TBK1 was regulated by heme. To address this issue, we used two mass spectrometry methods. The MALDI analysis revealed that the stoichiometry of phosphorylation of BACH2 IDR by TBK1 was not appreciably affected in the presence or absence of heme. In contrast, the LC-MS/MS analysis revealed that the phosphorylation sites of BACH2 IDR by TBK1 did change in the presence or absence of heme. We suggest that when heme is increased within cells, TBK1-BACH2 interaction is increased, leading to phosphorylation of heme-dependent serine and threonine residues of BACH2. This interpretation is supported by our previous findings that the structural state in the solution of BACH2 IDR changes in the presence of heme. Such alterations in structure may expose a specific set of serine and threonine for TBK1-mediated phosphorylation. When heme is not bound to BACH2 and TBK1 is activated by the cGAS-STING pathway,^79^^,^^80^ TBK1 may phosphorylate the none-heme-regulated sites. It has been previously reported that changes in phosphorylation sites within IDRs are responsible for the diversity of protein-protein interactions.^11^ Therefore, the phosphorylation of BACH2 IDR by TBK1 reduces the interactions of BACH2 with co-repressors and proteins important for its nuclear accumulation, leading to the induction of BACH2 target genes.

In B cells, BACH2 protein is present at around 7,500 molecules per cell in activated B cells but it is not detected in plasma cells.^81^ Our present findings suggest a molecular mechanism by which heme and phosphorylation act, in a cascade manner, to alter the conformational state of the BACH2 IDR and the entire BACH2 molecule, ultimately leading to dynamic alterations in its protein interactions and amount (Figure S6). How is this model reconciled with the heme- and FBXO22-mediated ubiquitination of BACH2? Considering that BACH2 binds multiple numbers of heme per molecule, the status of heme-binding may affect the pathways by which BACH2 is regulated (Figure S6). Under normal conditions, a few heme molecules may bind to BACH2, which allows its interaction with co-repressors like DNMT1 and NCOR1. When heme binding is increased, TBK1 is recruited to BACH2, phosphorylating BACH2 and thus reducing its interactions with those proteins. Under such conditions, FBXO22-mediated ubiquitination and degradation is not initiated yet and rather precluded. Inactive BACH2 would be retained for future recycling for gene repression. This state may also allow transcription activation by BACH2 via recruitment of co-activators. When heme binding is further increased, new conformational changes may be induced, leading to BACH2-FBXO22 interaction. Since BACH2-FBXO22 interaction was lost when multiple phosphorylation sites were mutated, further phosphorylation of BACH2 by TBK1 may also promote BACH2-FBXO22 interaction. To test this model, new methods such as a cryoelectron microscopy analysis of BACH2 and its binding proteins may be helpful. While multiple heme molecules bind to the BACH2 IDR, it is not clear how heme is delivered to BACH2. In a recent report, GAPDH has been identified as a heme chaperone that creates a bioavailable heme pool for transferring it to other proteins.^82^^,^^83^ Interestingly, GAPDH was also found in the ReCLIP analysis of BACH2 interacting proteins. Further investigation is needed to determine whether GAPDH functions as a heme chaperone for BACH2. It should be noted that the shorter BACH2 IDR fragment that did not contain CP motifs still bound to TBK1 in a heme-responsive manner (Figure S1). Since this region contains multiple histidine residues, heme may be coordinated by these residues. Further studies are required to understand the role of histidine residues for heme binding and TBK1 binding. Nonetheless, our present results clear show that heme regulates BACH2 protein in both CP-dependent and -independent manners.

In conclusion, our present study established heme-dependent protein-protein interactions of BACH2 via its IDR. It has been previously reported that heme acts as a signaling molecule to modulate gene expression.^31^^,^^84^ We found that heme is a signaling molecule of humoral immunity by changing BACH2 protein network; it recruits TBK1 to disrupt the interaction of BACH2 with the co-repressors and FBXO22. Importantly, multivalent binding of the BACH2 IDR and heme, together with TBK1-mediated phosphorylation, generated multiple stages of dynamic protein-protein interactions. Therefore, we believe that our findings provide insights for elucidating the function of various IDRs and regulating IDRs by artificial ligands toward drug development.

### Limitations of the study

While the *in vitro* binding assays using purified recombinant proteins clearly showed increases in the binding of BACH2 IDR with TBK1 or DNMT1 in response to heme, a quantitative measurement of binding parameters is necessary. Considering that FBXO22 binds to the BTB domain of BACH2, the effects of heme or phosphorylation upon BACH2-FBXO22 interaction appears to involve an overall structural rearrangement of BACH2. However, due to the presence of IDR, such changes are difficult to capture with currently available methods. It will also be important to validate the observed protein interactions mediated by BACH2 IDR in B cells.

## Resource availability

### Lead contact

Further information and requests for resources and reagents should be directed to and will be fulfilled by the lead contact, Kazuhiko Igarashi (kazuhiko.igarashi.a5@tohoku.ac.jp).

### Materials availability

Requests for resources and materials should be directed to the lead contact.

### Data and code availability

• Requests for primary data of proteomics should be directed to the lead contact.
• The paper does not report any original codes.
• Other information or items in this paper are available from the lead contact upon request.

## Acknowledgments

We thank Ms. R. Kitazawa and Dr. N. Ohsawa for technical assistance and Animal Experimentation and Biomedical Research Core, Tohoku University Graduate School of Medicine for technical support. This work was supported by a 10.13039/501100001691JSPS Grant-in-Aid for Scientific Research Number 19K06538 (M.W.-M.), 22H00443, 18H04021, and 15H02506 (K.I.); Research Fellowships for Young Scientists (M.W.-M., grant number 16J40189), and the 10.13039/100016925Takeda Foundation for the Promotion of Science and Research Grant in the Natural Sciences from the 10.13039/501100004398Mitsubishi Foundation.

## Author contributions

M.W.-M. conceived the study designed and performed most experiments. S.K., K.S., T.N., A.M., K.O., L.C.N., and K.D.-U. helped with some experiments. M.M. performed the bioinformatic analysis. H.S. and K.M. performed the mass spectrometry analysis. K.S. performed the mammalian two-hybrid assay. T.N. and K.N. performed and directed the ubiquitination assay. M.I. and M.S. prepared recombinant proteins for *in vitro* phosphorylation assays. K.M. and K.I. directed and coordinated the study and designed the research. M.W.-M. and K.I. wrote the manuscript with comments from all authors.

## Declaration of interests

K.I. and K.M. received partial financial support from Teijin Pharma Co., Ltd. through a collaborative research contract.

## STAR★Methods

### Key resources table

**Table:** 

REAGENT or RESOURCE	SOURCE	IDENTIFIER
**Antibodies**		

anti-BACH2-N2	Tanaka et al.^59^	Home made
anti-MafK	Oyake et al.^85^	Home made
anti-FBXO22	Santa Cruz Biotechnology	Cat #sc-100736
anti-TBK1/NAK	3504 Cell Signaling	Cat #3504
anti-DDDK-tag	MBL	Cat #M185-3L
anti-GAPDH	Abcam ab8245	Cat #ab8245
anti-Lamin B(C-20)	Santa Cruz Biotechnology	Cat #sc-6216
anti-p70S6K	Cell Signaling Technology 9202	Cat #9202
anti-phospho-p70S6K	Cell Signaling Technology 9205	Cat #9205
anti-GST	Sigma Aldrich	GE27–4577-01
anti-HA	Sigma-Aldrich	N/A
anti-DNMT1	Abcam	Cat #ab13537
Goat anti-Rabbit IgG secondary antibody HRP	Thermo Fisher Scientific	Cat #31460
Goat anti-Mouse IgG (H + L) Secondary Antibody, HRP	Thermo Fisher Scientific	Cat #31430
Donkey anti-Rabbit IgG (H + L) secondary antibody, Alexa Fluor™ 488	Thermo Fisher Scientific	A-21206
Donkey anti-Goat IgG (H + L) secondary antibody, Alexa Fluor™ 555	Thermo Fisher Scientific	A-21432

**Bacterial and virus strains**		

E. coli: Strain: KRX	Promega	L3002

**Biological samples**		

His-FLAG-DNMT1	BPS Bioscience	Cat#51100

**Chemicals, peptides, and recombinant proteins**		

FLAG peptide	Merck	F3290
BX795	CEM	CS-0259
MRT67307	Merck	506306
Dithiobis[succinimidyl propionate] (DSP)	Thermo Fisher Scientific	PG82081
Dithio-bismaleimidoethane (DTME)	Thermo Fisher Scientific	22335
cOmplete™, EDTA-free Protease Inhibitor Cocktail	Roche	04693132001
PhosSTOP™ phosphatase inhibitor	Roche	4906845001
Trypsin Gold	Promega	V5280
FuGENE® HD Transfection Reagent	Promega	E2311
GeneJuice® Transfection Reagent	Merck	70967
PEI MAX®	Polysciences	24765
TransFectin Lipid Reagent	Bio-Rad	1703350
Kinase Buffer (10X)	Cell Signaling Technology	9802
IMDM for SILAC	Thermo Fisher Scientific	88367
Dialyzed FBS for SILAC	Thermo Fisher Scientific	89986
L-Lysine-^13^C_6_, hydrochloride	FUJIFILM Wako	120–06091
L-arginine-HCl-^13^C_6_^15^N_4_, hydrochloride	FUJIFILM Wako	010–24041

**Critical commercial assays**		

HA-tagged Protein Purification Kit	MBL	3342
RNeasy micro kit	QIAGEN	74004
Dual-luciferase® Reporter Assay System Dual-Glo® Luciferase Assay System	Promega Promega	E1910 E2920
B cell isolation kit mouse	Miltenyi Biotec	130-090-862
Bac-to-BacT^M^ Vector kit	Life Technologies	10712024
SuperScript™ III Reverse Transcriptase	Thermo Fisher Scientific	18080093
FastStrand DNA Master SYBR Green I FastStrand DNA Master SYBR Green I	Roche	03003230001

**Experimental models: Cell lines**		

mature B cells: BAL17	Ando et al.^66^	N/A
a leukemia cell line of myeloid precursor cells: M1 cell	Ebina-Shibuya et al.^86^	N/A
HEK293T	Ando et al.^66^	N/A
PlatE packaging cells	Morita et al.^87^	N/A

**Experimental models: Organisms/strains**		

Mouse: Blimp-1_EGFP transgene	Muto et al.,^36^ Ohinata et al.^58^	N/A
Mouse: BACH2-deficient mice	Muto et al.^35^	N/A

**Oligonucleotides**		

Primers of RT-PCR for Pre-mRNA Prdm1: mouse Pre-mRNA_Prdm1 F: GGCTGTCTTTCACACTGCATCT	This paper	N/A
Primers of RT-PCR for Pre-mRNA Prdm1: mouse Pre-mRNA_Prdm1 R: ACGTAGCGCATCCAGTTG	This paper	N/A
Primers of RT-PCR for TBK1: mouse TBK1 F: GGTGCACTATGCCGTTCTCT	This paper	N/A
Primers of RT-PCR for TBK1: mouse TBK1 R: TGTTCTAGAGGAGCCGTCCA	This paper	N/A
Primer of ChIP qPCR for TBK1: mouse TBK1 #1 MARE Chip 1F: ACTCCCTGGGTAGGTGGTTC	This paper	N/A
Primer of ChIP qPCR for TBK1: mouse TBK1 #1 MARE Chip 1R: CACGTCAGTCTTGAGGGCAG	This paper	N/A
Primer of ChIP qPCR for TBK1: mouse TBK1 #2 MARE Chip 2F: TGGTCTGAAACTTTTCCAGGA	This paper	N/A
Primer of ChIP qPCR for TBK1: mouse TBK1 #2 MARE Chip 2R: CAGAATCACAGATAACTTACTTGTTGAATG	This paper	N/A
Primer of ChIP qPCR for MCM5: mouse MCM5 F: GCGAAAGTCGGCTTCCTCTA	This paper	N/A
Primer of ChIP qPCR for MCM5: mouse MCM5 R: CAATTCCCTCACCTCACAGC	This paper	N/A
siFbxo22-1 targeting sequence: TCTCGTTCACCTGGGAACCTCTAAA	This paper	N/A
siFbxo22-2 targeting sequence: GGGAATTGTGGTGACTCCAATGGGA	This paper	N/A
Stealth RNAi siRNA Negative Control Med GC	Thermo Fisher Scientific	12935300

**Recombinant DNA**		

pOZ-FH-N BACH2 331-520	This paper	N/A
pGL4-4.28 [luc2cp miniP] *Slc48a1*	This paper	N/A
*Human TBK1* cDNA	Takara Bio INC	cDNA (NM_013254)
pFN21KB0999: human *NCOR2*	This paper	Accession No. AB463148
pFN21AA1047: human *NCOR1*	This paper	Accession No. AB385422
*Human BACH2* cDNA	Takara Bio INC	cDNA (NM_021813)
pFN26AhBACH2BTB	This paper	N/A
Human *FBXO22* cDNA	FASMAC	cDNA (NP_671717)
His-CDC34	Nakagawa et al.^88^	N/A
His-UbcH5A	Nakagawa et al.^88^	N/A

**Software and algorithms**		

BZ-H4C software program	KEYENCE	N/A
Qual Browser software	Thermo Fisher Scientific	N/A
Mascot	Matrix Science	N/A
Integrative Genomics Viewer (IGV v2.11.1)	Robinson et al.^55^	N/A

### Experimental model and study participant details

The mice carrying the *Blimp-1_EGFP* transgene and the *Bach2*-deficient mice were reported previously.^35^^,^^36^^,^^58^ All the experiments involving mice were approved by the Institutional Animal Care and Use Committee of the Tohoku University Environmental & Safety Committee. Mice were housed at the Institute of Animal Experimentation, Tohoku University Graduate School of Medicine, under specific pathogen free conditions and *ad libitum* feeding. Both male and female mice between 8 and 12 weeks old were used in this study. There were no notable differences observed in plasma cell differentiation *in vitro* between B cells derived from male and female mice. This study did not involve any human participants.

### Method details

#### Plasmid and transfection

To construct pGEX6P1-BACH2 381–481, pGEX6P1-BACH2-IDR plasmid was used as the template for PCR amplification with primers:5′-CTTAGGATCCAAAACTGATTACACCCCTTTGGC-3′, which introduced a BamHI site; and 5′-CCGCGGCCGCTTATCAGTGGGAGTAAGCCTGGGA-3′, which introduced a NotI site adjacent to the termination codon (Invitrogen). The amplified fragment was then digested with BamHI and NotI, and cloned into the pGEX-6P-1 vector (Cytiva Global Life Sciences Solutions), resulting in pGEX6P1-BACH2-381–481. The pCMV BACH2, pEF MAFK and pEF seapansy were described previously.^85^ The retrovirus vector pOZ-FH-N BACH2-331–520 containing the cDNA corresponding to amino acid residues 331–520 of mouse BACH2 was generated by PCR from pcDNA FLAG3.1 BACH2^89^ using a SalI site-containing forward primer and NotI site-containing reverse primer: 5′-CGTGTCGACTCCAGGAGTGTGTCCTCGCCT-3′, which introduced a SalI site; and 5′-CCGCGGCCGCTTATCAGCTGGAGGTCCTAGT-3′, which introduced a NotI site adjacent to the termination codon (Invitrogen). The amplified fragment was digested with XhoI and NotI (New England Biolabs) and was cloned into the pOZ-FH-N plasmid,^46^ resulting in pOZ-FH-N-BACH2-331–520. The full-length mouse TBK1 was cloned into a modified pFastBac vector with an N-terminal FLAG-8xHis-tag and HRV 3C protease site. A recombinant bacmid DNA was generated using the Bac-to-Bac system (Life Technologies). The reporter containing the intron MARE of *Slc48a1*, Hrg1 gene (intron-MARE-luc) was generated by PCR from C57/BL6J genomic DNA using the following primers: 5′- GGAGGTACCAGGCAGTCACCCT-3′, which introduced a KpnI site; and 5′- GGACTCGAGCCCAGATGCTCAT-3′, which introduced an XhoI site. The 220-bp PCR fragment containing the intron MARE was inserted into pGL4-4.28 [luc2cp miniP]. Both pCMVBACH2 and pEFseapansy have been described previously.^46^ pcDNA FLAG3.1mouseTBK1 was generated by PCR from C57/BL6J genomic DNA using the following primers: 5′-AAGAATTCATGCAGAGCACCTCCAACCAT-3′, which introduced an EcoRI site; and 5′-AAGGTACCTTATCACTAAAGACAGTCCACATTGCGAAGG-3′, which introduced a KpnI site. The pFN21AA1047 and pFN21KB0999 plasmids, which contains the human *NCOR1* and *NCOR2* ORFs (Accession No. AB385422 and AB463148), respectively, were purchased from Promega and were cloned into the pFN10A vector (Promega) using the SgfI and PmeI sites. These plasmids were named pFN10AhNCOR1 and pFN10AhNCOR2. The human *TBK1* cDNA (NM_013254) was generated by PCR from human reference cDNA (Takara Bio) and was cloned into pHTN (Promega) and was subcloned into pCAG-neo (FUJIFILM) using the BamHI and NotI sites. This plasmid was named pCAGhTBK1. The human *BACH2* cDNA (NM_021813) was generated by PCR from human thymus cDNA (Takara Bio) and was cloned into pCR-Blunt2-TOPO (Thermo Fisher Scientific) and was subcloned into pFN26A (FUJIFILM) using SgfI and PmeI. This plasmid was named pFN26AhBACH2. To construct pFN26AhBACH2-BTB (amino acids 1–128), the pFN26AhBACH2 plasmid was used as the template for PCR amplification. The amplified fragment was then digested with SgfI and PmeI, and was cloned into the pFN26A vector. The human *FBXO22* cDNA (Accession No. NP_671717) was synthesized by FASMAC (Kanagawa, Japan) and was cloned into the pFN10A vector (Promega) using the SgfI and PmeI sites, which was named pFN10AhFBXO22. To construct pcDNAFLAG-BACH2-HR-5 (S352A,S357A,T382A,S402A,S472A), pcDNAFLAG-BACH2-NHR-5 (S342A, T347A, S352A, S464A, S472A), pcDNAFLAG-BACH2-All (S342A, T347A S352A, S357A,T382A,S402A, S464A, S472A), we used a site-directed mutagenesis system (Agilent). To generate recombinant retroviruses, PlatE packaging cells^87^ were transfected with the pOZ-FH-C, pOZ-FH-C-BACH2^66^ and pOZ-FH-N-BACH2-331-520 vectors using the FuGENE HD transfection reagents (Promega), and then the supernatants were recovered 2 and 3 days after transfection, which were used to infect BAL17 mature B cells.

#### Cell culture

Mouse splenic B cells were cultured in RPMI 1640 medium (Sigma-Aldrich) supplemented with 10% FBS (Nichirei Biosciences, Japan), 10 mM HEPES (Thermo Fisher Scientific), 1 mM sodium pyruvate (Thermo Fisher Scientific), 0.1 mM non-essential amid acids (Thermo Fisher Scientific), 50 μM 2-mercaptoethanol (Wako), 100 μg/mL streptomycin (Thermo Fisher Scientific), and 100 units/ml penicillin (Thermo Fisher Scientific) as described.^66^ Splenic B cells were isolated from 8- to 12-week-old wild type C57BL/6, *BACH2* KO or Blimp-1_EGFP mice using a B cell isolation kit (Miltenyi Biotec) and cultured in RPMI 1640 medium as described previously.^36^ For the experiments using the kinase inhibitor, splenic B cells were cultured with 1 μM BX795 (Sigma-Aldrich) for 3 h, 1 day or 2 days. BAL17 mature B cells, M1 cells and HEK293T cells were maintained as described previously^66^^,^^86^ and were tested regularly for mycoplasma contamination using e-Myco plus Mycoplasma PCR Detection Kit (iNtRON Biotechnology). The cell lines were used in our previous reports^66^^,^^85^^,^^86^ and were not authenticated by third parties.

#### FLAG-HA-BACH2-IDR protein purification

The FLAG- and hemagglutinin (HA)-epitope-tagged BACH2-331–520 protein fragment (denoted as FLAG-HA-BACH2-IDR) was purified from whole cell extracts prepared from BAL17 mature B cells stably expressing FLAG-HA-BACH2-331-520 by using the ReCLIP method.^49^ Briefly, in-cell cross-linking was performed using Dithiobis[succinimidyl propionate] (DSP) and Dithio-bismaleimidoethane (DTME) (Thermo Fisher Scientific). After removal of the buffer containing the cross-linkers, cells were incubated at 25°C for 10 min with a quenching solution (20 mM Tris-HCl pH 7.4, 5 mM L-Cysteine). The quenching solution was then removed and cells were lysed with RIPA buffer (50 mM Tris-HCl (pH 7.4), 150 mM NaCl, 1% NP-40, 0.5% deoxycholic acid sodium salt, 0.1% SDS, 1 × protease inhibitor (Roche Ltd. Basel, Switzerland), and 1 × Phos STOP (Roche). The lysate was incubated with FLAG-M2 agarose beads (Sigma-Aldrich) for 2 h at 4°C. The beads were washed three times with RIPA buffer. The bound proteins were eluted from the FLAG-M2 agarose beads by incubation in a wash buffer including 0.15 mg/mL 5× FLAG peptide (Sigma-Aldrich) for 30 min at 4°C. To reverse the cross-link of proteins, the eluted protein solutions were incubated with 50 mM DTT for 30 min at 37°C and then with an SDS-PAGE sample buffer for 5 min at 95°C. The proteins were subjected to SDS-PAGE using a 5–20% gradient gel, and the gel was stained with Coomassie Brilliant Blue. The gel was subjected to in-gel reduction with 10 mM DTT and in-gel alkylation with 55 mM acrylamide. These samples were then digested with trypsin. The peptides were ionized and were sprayed directly into an LTQ-Velos Orbitrap with ETD mass spectrometer (Thermo Fisher Scientific). The full mass scans were acquired with the Orbitrap.

#### Expression and purification of BACH2-IDR and His-FLAG-TBK1

Expression and purification of recombinant BACH2-331-520 (331–520 amino acid region) and GST-BACH2 --381–481 (amino acid region 381–481) in an *E. coli* expression system was carried out as described previously.^47^ Briefly, *Escherichia coli* KRX cells (Promega, Southampton, UK) were used for expression which was induced with 0.2 mM IPTG. After sonication of the cells, the extract was applied to Glutathione Sepharose 4B column (GE Healthcare Life Sciences) and the recombinant protein was eluted using 10 mM reduced glutathione. Depending on the purpose of experiments, the GST tag was removed (BACH2-IDR) or was kept (GST-BACH2-IDR). Recombinant mouse His-FLAG-TBK1 was expressed in Sf9 insect cells. Baculovirus was generated by transfecting purified bacmid DNA into Sf9 cells using FuGENE HD (Promega) and was subsequently used to infect suspension cultures of Sf9 cells (2×10^6^ cells/ml) with a multiplicity of infection of 1. The infected Sf9 cells were incubated at 27°C for 48 h for protein expression. The Sf9 cells were suspended in buffer A (20 mM Tris-HCl, pH 8.0, 150 mM NaCl, 10% glycerol, and 1× protease inhibitor), were sonicated at 4°C and were centrifuged at 28,980 × *g* for 20 min. Soluble protein fractions were mixed with FLAG-M2-agarose beads (Sigma-Aldrich) pre-equilibrated with buffer A. The His-FLAG-TBK1 protein was eluted using buffer A containing 3×FLAG peptide (200 μg/mL), was concentrated using an Amicon Ultra-15 centrifugal filter unit (Merck, Billerica, MA), and was applied to a G-25 spin column (Cytiva) pre-equilibrated with a buffer including 20 mM Tris-HCl pH 8.0, 150 mM NaCl, 1 × protease inhibitor, and 1 × phosstop. Recombinant human His-FLAG DNMT1 was purchased from BPS Bioscience.

#### Pull-down of GST-BACH2-IDR using BAL17 extract

GST-BACH2IDR was prepared as above^47^ and was applied to a PD-10 column (Cytiva) pre-equilibrated with a buffer including 20 mM HEPES, pH 7.0, 50 mM NaCl, and 2 mM TCEP to change the buffer. BAL17 whole cell extracts were prepared as described previously.^59^ In brief, one hundred million of BAL17 cells were lysed in Buffer C (20 mM HEPES pH7.5, 20% glycerol, 400 mM NaCl, 1 mM EDTA, 1 mM MgCl_2_, 0.5 mM PMSF, 0.1% Nonidet P-40, 1 × protease inhibitor (Roche), and 1 × Phos STOP (Roche). The buffer of the BAL17 whole cell lysate was changed as above. The prepared BAL17 whole lysates and 2 μg of GST-BACH2-IDR were mixed in the presence or absence of 0.05 μM heme and then the protein mixtures were incubated with Glutathione Sepharose beads (Cytiva) for 2 h at 4°C. The beads were washed three times in a wash buffer including 20 mM HEPES, pH 7.0, 50 mM NaCl, and 5 mM DTT. The bound proteins were eluted in an elute buffer including 20 mM HEPES, pH 7.0, 50 mM NaCl, 2 mM DTT, and 10 mM glutathione. The protein samples were next prepared using an SDS-PAGE sample buffer. GST-BACH2IDR, TBK1 and DNMT1 were detected by immunoblotting using anti-GST, anti-TBK1 or anti-DNMT1 antibodies.

#### Pull-down of GST-BACH2-IDR, His-FLAG-TBK1 and His-FLAG-DNMT1 proteins

The recombinant proteins of GST-BACH2-IDR (2 μg: 0.05 μM) or GST-BACH2-381–481 (2 μg: 0.05 μM), His-FLAG-DNMT1 (1 μg: 0.02 μM) and His-FLAG-TBK1 (1 μg: 0.015 μM) expressed and purified as described above were incubated with Glutathione Sepharose beads (Cytiva) in the presence or absence of 0.025 μM or 0.05 μM heme for 2 h at 4°C. The beads were washed three times in a buffer including 20 mM HEPES pH 7.0, 50 mM NaCl, 2 mM TCEP. The bound proteins were eluted in the same buffer containing 10 mM glutathione and were analyzed by SDS-PAGE and immunoblotting as above.

#### Immunofluorescence microscopy analysis

M1 cells were placed on microscope cover glasses (MATSUNAMI) and were incubated with or without 5 μM BX795 for 2 h. The cells were fixed with 4% formaldehyde (Merck) in PBS for 10 min. After being washed with PBS three times, cells were incubated with 0.5% Triton X-100 in PBS for 5 min. After that, cells were incubated with a purified anti-BACH2-N2 antibody and anti-Lamin B antibody (M-20) for 1 h. Donkey anti-rabbit IgG labeled with Alexa Fluor 488 (Invitrogen) or donkey anti-goat IgG labeled with Alexa Fluor 555 (Invitrogen) were used as secondary antibodies. Nuclei were stained with 10 μM Hoechst 33342 (Sigma-Aldrich). Images were collected using a BZ-X800 microscope (KEYENCE). Images were analyzed by a BZ-H4C software program (KEYENCE).

#### Preparation of whole cell extract

M1 cells (1×10^7^) were incubated with or without 5 μM BX795 for 1 h. M1 cells were lysed in RIPA buffer and were centrifuged at 20,400 × *g* for 10 min. Each sample was mixed with the SDS-PAGE sample buffer and then was heated for 5 min at 95°C. BAL17 and mouse splenic B whole cell extracts were prepared as above. The cells were lysed in Buffer C and were centrifuged at 20,400 × *g* for 10 min. Each sample was mixed with an SDS-PAGE sample buffer and then was incubated for 5 min at 95°C.

#### Subcellular fractionation

Eight million mouse splenic B cells were lysed in 100 μL of a lysis buffer (PBS, 0.1% NP-40, 1 × protease inhibitor (Roche Ltd.), and 1 × Phos STOP (Roche)). The resulting solution was divided equally into two, and one-half (50 μL) was saved as the whole cell extract and the other (50 μL) was next centrifuged at 800 × *g* for 1 min. The supernatant solution was saved as the cytosol fraction. The precipitation of this sample was washed with ice-cold PBS three times and then was dissolved in 50 μL of the lysis buffer and was saved as the nuclear fraction. All the subcellular fractions were mixed with an SDS-PAGE sample buffer and then were incubated for 5 min at 95°C.

#### Immunoblot analysis

The samples were resolved by SDS-PAGE and was electro-transferred onto PVDF membranes (Merck). The membranes were blocked for 1 h according to the requirements of the primary antibodies used. The membranes were subsequently incubated with primary antibodies in a blocking buffer (3% nonfat dry milk and Tris-buffered saline with Tween 20) overnight at 4°C. The membranes were washed and were incubated with secondary antibodies for 30 min. The primary antiserum and antibodies used were an anti-BACH2 anti-serum (BACH2-N2), and anti-TBK1/NAK (3504, Cell Signaling Technology), anti-DNMT1 (ab13537, Abcam), anti-GST (GE27–4577-01, Sigma Aldrich), anti-phospho-p70S6K (9205, Cell Signaling Technology), anti-p70S6K (9202, Cell Signaling Technology), anti-Lamin B (C-20) (sc-6216, Santa Cruz Biotechnology) and anti-GAPDH antibodies (ab8245, Abcam). ECL Western blotting detection reagents (Cytiva) were used to detect antibody-antigen complexes.

#### Chromatin immunoprecipitation

Chromatin fixation and immunoprecipitation were carried out using BAL17 cells and splenic B cells as described previously using BACH2-N2 antibody.^59^^,^^90^ In brief, cells were fixed by adding formaldehyde to 1% final concentration for 10 min at room temperature. Cells were then sonicated to prepare chromatin suspensions of roughly 300- to 1,000-bp DNA in length. Immunoprecipitations were carried out as described.^59^ Relative immunoprecipitation efficiency was calculated from the ratio between immunoprecipitated sample and input. Primers used to detect *Hmox1 E1 and E2* regions were previously reported.^91^ Primers used to detect promoter regions of *Mcm5*: Forward 5′-GCGAAAGTCGGCTTCCTCTA-3′, Reverse 5′-CAATTCCCTCACCTCACAGC-3′and *Slc48a1*: Forward 5′-CTTCCTCTTTTAGGGTGGAATGT, Reverse 5′-GATGCTCATACATACCGTGTTCC. The primers used to detect the *Prdm1* promoter and intron 5 MARE regions were previously described.^59^ Other primers used to detect the #1 and #2 sites of *Tbk1* are shown in key resources table.

#### Electrophoretic mobility shift assay

Each probe was labeled with [γ-^32^P]-ATP (PerkinElmer) using T4 polynucleotide kinase (Takara Bio). The double-stranded probes were as follows:

TBK1#1_WT 5′-TAGCCTGTGCTGATTCAGCAAGCCGT-3'; TBK1#1_Mut, 5′- TAGCCTGGAGATAAATCCCAAGCCGT-3'; TBK1#2_WT 5′-GACTAAAACATGACTCAGCACACCCAACG-3'; TBK1#2_Mut 5′- GACTAAAAGAGATAAATCGGGACCCAACG-3'.

*Slc48a1*-MARE, 5′-GCACTCCACTTCTGACTCAGCATTTTGCAGCC-3′; *Slc48a1*-mut-MARE, 5' -GCACTCCACTGAGATAAATCATTTTGCAGCC-3′

The underline represents the potential MARE sequences. For the competition and super-shift analyses, GST-BACH2_331–839 (amino acids 331–839) was incubated for 30 min with the non-labeled double-stranded oligo DNAs (TBK1#1_WT, TBK1#1_Mut, TBK1#2_WT or TBK1#2_Mut) as competitors, a NRS, antiserum against BACH2 (F69-1)^85^ or antiserum against MafK (A-1).^92^ The resulting reaction solutions were separated by electrophoresis through a 4% polyacrylamide gel, which was then dried and exposed to a BAS-MS2040 imaging plate (FUJIFILM), followed by detection with BAS1500 (FUJIFILM). Expression and purification of GST-BACH2_331–839 and MBP-MafK were carried out as described previously.^32^^,^^85^^,^^92^ In brief, the GST fusion was purified from Escherichia coli Rosetta (DE3) cells (Merck) transformed with the expression construct using glutathione Sepharose 4B and heparin-sepharose CL-6B columns (GE Healthcare). The MBP fusion was purified using amylose resin (New England Biolabs).

#### Quantitative PCR with reverse transcription (RT-qPCR)

RNA was isolated with an RNeasy micro kit (QIAGEN) and was converted to cDNA with random primers and a Superscript III reverse transcriptase (Invitrogen). Light Cycler FastStrand DNA Master SYBR Green I (Roche) reagents and a Light Cycler system (Roche) were used for qPCR. PCR primer of *Slc48a1* (HRG-1) were as follows: *Slc48a1* Forward:5′- TTCTTCTTCGTGGGTGCTCT-3′ and *Slc48a1* Reverse:5′- AGCTGTTCGGGTCTTTGAGA -3’.

#### Luciferase reporter assay

HEK293T cells were transfected with various combinations of the reporter and effector plasmids using the GeneJuice Transfection Reagent (Merck) according to the manufacturer’s instructions. Each transfection was done in duplicate, and the luciferase activity was measured 24 h after transfection using the dual-luciferase reporter assay kit (Promega) according to the manufacturer’s protocol. Normalized values are reported as the mean ± standard deviation (SD) from three independent experiments.

#### Flow cytometry (FACS)

The cells were analyzed in a FACSVerse flow cytometer (BD Biosciences).

#### Mammalian two-hybrid assays

HEK293Tcells were plated onto a 96-well D Lysine plate (CONING) at a density of 2×10^4^ cells per well in DMEM containing 10% FBS and 4 mM glutamine. After 24 h of cell culture, 10 ng of plasmids of pGL4.35-9×GAL4UAS-Luc (Promega), pFN26AhBACH2, pCAG-neo-hTBK1, pFN10AhNCOR1 or pFN10AhSMRT were transfected for each condition. using the Lipofectamin LTX with PLUS reagent (Thermo Fisher Scientific). At 24 h after transfection, cells were treated with 0.1 or 1.0 μM TBK1 inhibitor (MRT67307, Merck) for 6 h. Cell extracts were analyzed with the Dual-Glo Luciferase Assay System (Promega) using a SpectraMax Paradigm (Molecular Devices). Samples were run in quadruplicate, and the SD were calculated. Data are representative from at least 3 independent transfections.

#### *In-vitro* kinase assay

Purified BACH2IDR (10 μM) and His-FLAG-TBK1 (0.5 μM) were used for the assay. The reactions were performed at 30°C using a Kinase Buffer (#9802, Cell Signaling Technology), supplemented with 0.1 mM ATP, supplemented with protease inhibitor (Roche) and phosphoStop^93^ at 30°C. Heme was added in the kinase reaction at 5 or 10 μM for the time-course experiment (0, 2, 4, 6, 8, 10, 15, 20 min). Kinase reactions were stopped by the addition of an SDS-PAGE sample buffer containing either 0.5% TFA (LC-MS/MS) or 0.1% TFA (MALDI-TOF experiment).

#### Stable isotope labeling using amino acids in cell culture analysis

Iscove’s modified Dulbecco’s medium for SILAC (Thermo Fisher Scientific) containing 10% dialyzed FBS (Thermo Fisher Scientific) and 2-mercaptoethanol (FUJIFILM Wako) was used by adding either 0.4 mM L-lysine-HCl (Merck) with 0.2 mM L-arginine-HCl (Merck) as the “light” medium or 0.4 mM L-lysine-HCl-^13^C_6_ and 0.2 mM L-arginine-HCl-^13^C_6_^15^N_4_ (FUJIFILM Wako) as the “heavy” medium.^66^ Before collection of cells, the light-labeled cells were transferred to SILAC light medium without FBS containing 5 μM heme (Merck) and were incubated for 4 h. Each sample of the heavy-labelled cells (around 1 × 10^8^ cells) was mixed with the equal number of the light-labelled cells and was suspended in 2 mL of a lysis buffer (50 mM Tris-HCl, pH 7.4, 150 mM NaCl, 0.3% Nonidet P-40, cOmplate (Roche) and PhosStop (Roche)). After centrifugation at 20,400 × g for 10 min at 4°C, the resulting cleared cell lysate was mixed with anti-FLAG magnetic beads (Sigma M8823, 40 μL of the slurry was used for a sample) with gentle rotation for 2 h at 4°C. The beads were washed three times using 0.5 mL of the lysis buffer. The proteins bound to the beads were eluted in 40 μL of the lysis buffer containing 0.2 μg/mL FLAG peptide (F3290, Merck). The samples were separated by SDS-PAGE using a 5–20% gradient gel (HOG-0520, ORIENTAL INSTRUMENTS). After reduction with DTT, alkylation using acrylamide and tryptic digestion,^88^ the samples were subjected to LC-MS/MS as described in a previous report.^66^

#### *In-vitro* ubiquitination assay

Ubiquitination assays were performed in 21 μL reaction volume containing the following components: 10 μg ubiquitin, 200 ng His-CDC34, 100 ng His-UbcH5A, 60 ng HA-BACH2, 100 ng SCF^Fbxo22^, 0.5 U creatine phosphokinase, 1 mM phosphocreatine and 2.1 μL of a 10× reaction buffer consisting of 250 mM Tris-HCl (pH 7.5), 1.2 M NaCl, 20 mM ATP, 10 mM MgCl_2_, 50 μM ZnCl_2_ and 10 mM DTT. The reaction mixtures were incubated at 37°C for 90 min before the reaction was terminated by the addition of the SDS-PAGE sample buffer, and were immediately heated to 95 °C for 5 min. Proteins were separated by SDS-PAGE and then were immunoblotted with an anti-HA antibody (Sigma-Aldrich). Expression and purification of ubiquitin, His-CDC34, His-UbcH5A and SCF^Fbxo22^ were carried out as described previously.^94^ In brief, His-UbcH5A was expressed using pET30a. HA-BACH2 was expressed and was purified from HEK293T cells; HEK293T cells were plated onto 15 cm dishes (4 dishes) at a density of 6∼7×10^6^ cells per dish in DMEM containing 10% FBS. After 24 h of incubation, cells were transfected with 15 μg of pcDNA3-HA-BACH2 per dish using the PEI MAX reagent (Polysciences). At 48 h after transfection, cells were lysed with an NP-40 buffer (50 mM Tris pH 7.5, 150 mM NaCl, 0.5% Nonidet P-40, 10% Glycerol, and a protease inhibitor cocktail (10 μg/mL aprotinin, 10 μg/mL leupeptin, and 1 mM phenylmethylsulfonyl fluoride)) and then HA-BACH2 was purified from the resulting lysates with the use of an HA-tagged Protein Purification Kit (MBL).

#### MALDI-TOF mass spectrometry

The reaction solutions of *in vitro* kinase assays were sampled (1 μL aliquots) over 30 min and were diluted in 10 μL of distilled water. Subsequently, the samples were spotted onto a MALDI sample plate with 1 μL of sinapinic acid (Sigma-Aldrich) dissolved in a solution of 10 mg/mL in 0.1% TFA and 50% acetonitrile as the matrix. Each spot was analyzed with an AXIMA Performance MALDI-TOF mass spectrometer (Shimadzu, Kyoto, Japan). The spectra were monitored in the linear positive mode within a mass range of 19,500 to 22,500 Da. The mass shifts in the time-course kinase reactions were evaluated using the spectrum of BACH2 IDR (molecular weight 20533.04) at the reaction time of 0 min as the standard.^66^^,^^74^

#### Liquid chromatography-tandem mass spectrometry

*in vitro* kinase assay samples were treated with DTT, acrylamide and tryptic digestion in the solution. Phosphopeptides were enriched by TiO_2_ affinity purification (GL Sciences),^66^^,^^95^ and were subjected to LC-MS/MS using an Orbitrap Fusion mass spectrometer equipped with an Easy-nLC 1000 HPLC system (Thermo Fisher Scientific). The peptides were separated by reversed-phase chromatography using a C18 tip column (75-μm ID × 10-cm L, Nikkyo Technos), 0.1% formic acid in water as the aqueous solvent and 0.1% formic acid in acetonitrile as the organic solvent and were electrosprayed into the mass spectrometer for MS/MS acquisition. Peptide identification was done using a MASCOT search engine by searching the *E*. *coli* proteins in the Swissprot database and a homemade protein list including the mouse BACH2 and human TBK1 proteins as well as common contaminating proteins. Propionamidation at Cys residues was chosen as the fixed modification and acetylation at protein N-term, oxidation at Met and phosphorylation at Ser/Thr were considered as the variable modifications. For quantification of phosphopeptide, extracted ion chromatograms of the *m*/*z* value of the precursor ion were obtained from all the raw MS/MS data, irrespective of whether the phosphopeptide was detected in every sample, and the peak area values were calculated using a Qual Browser software (Thermo Fisher Scientific).

#### FLAG immunoprecipitation

HEK293T cells were transfected with either the wild-type or mutant BACH2 plasmid using the TransFectin Lipid Reagent (BIO-RAD) according to the manufacturer’s instructions.

After 24 h, each sample of cells (around 5 × 10^7^ cells) was collected and was suspended in 0.6 mL of a lysis buffer (50 mM Tris-HCl, pH 8.0, 120 mM NaCl, 0.1% Triton X-100, cOmplate (Roche) and PhosStop (Roche)), was sonicated at 4°C and was centrifuged at 20,400 × g for 10 min at 4°C. The resulting cleared cell lysate was mixed with anti-FLAG magnetic beads (Sigma M8823, 20 μL of the slurry was used for a sample) with gentle rotation for 2 h at 4°C. The beads were washed three times using 0.5 mL of the lysis buffer. The proteins bound to the beads were eluted in 20 μL of the lysis buffer containing 0.2 μg/mL FLAG peptide (F3290, Merck). The sample was separated by SDS-PAGE using a SuperSep Ace, 5–20% gel (FUJIFILM, Wako). FLAG-BACH2, TBK1 and FBXO22 were detected by immunoblotting using anti-FLAG, anti-TBK1, anti-MafK (homemade) and anti-FBXO22 antibodies.

#### Mouse FBXO22 knockdown

Stealth RNAi duplexes against FBXO22 were designed using the BLOCK-iT RNAi Designer (Invitrogen). We also used Stealth RNAi siRNA negative control (Invitrogen) that is not homologous to any sequence of the vertebrate transcriptome. For delivering RNAi duplexes, 3 × 10^6^ mouse splenic B cells were transfected with 6 μL of stock Stealth RNAi duplexes (20 nM) using a basic nucleofection solution for mouse primary B cell (VPA-1010; Lonza) with the nucleofector program (Z-001; Lonza). The oligonucleotide sequences of short hairpin RNA directed against FBXO22 mRNA were described in key resources table. The splenic B cells were stimulated with LPS (20 μg/mL) and incubated for 24 h. After that, the cells were lysed in RIPA buffer and were centrifuged at 20,400 × *g* for 10 min. Each sample was mixed with the SDS-PAGE sample buffer and then was heated for 5 min at 95°C.

### Quantification and statistical analysis

The data are presented as the means ± SD of at least triplicate determinations. The statistical analyses were performed by the use of the Student’s t test. R and Excel were used for statistical analyses.

## Supplemental information

Document S1. Figures S1–S6

Table S1. List of protein and their SILAC-ratio (Heavy/Light<1), related to Figure 4

Table S2. List of protein and their SILAC-ratio (Heavy/Light>1), related to Figure 4
