# Supplementary material for: Heme regulates protein interactions and phosphorylation of BACH2 intrinsically disordered region in humoral response
Source: iScience. 2024 Dec 4;28(1):111529. doi: 10.1016/j.isci.2024.111529 (PMC11699347; doi:10.1016/j.isci.2024.111529)
Supplement: Document S1. Figures S1–S6 [file mmc1.pdf]

iScience, Volume *28*

Supplemental information

Heme regulates protein interactions and phosphorylation of BACH2 intrinsically disordered region in humoral response

Miki Watanabe-Matsui, Shun Kadoya, Kei Segawa, Hiroki Shima, Tadashi Nakagawa, Yuko Nagasawa, Shuichiro Hayashi, Mitsuyo Matsumoto, Mariko Ikeda, Akihiko Muto, Kyoko Ochiai, Long C. Nguyen, Katsumi Doh-Ura, Mikako Shirouzu, Keiko Nakayama, Kazutaka Murayama, and Kazuhiko Igarashi

Supplemental information figures and legends

SUPPLEMENTAL FIGURES

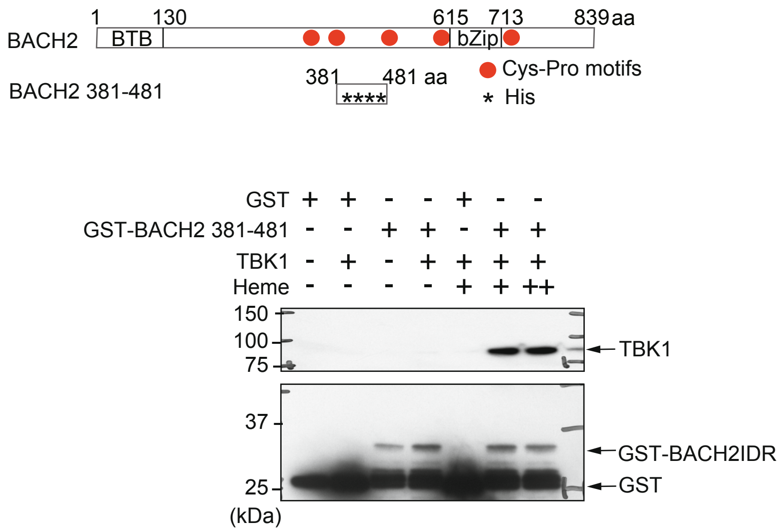

Figure S1, related to Figure 1

Figure S1. Pull-down of GST-BACH2 381-481 and His-FLAG TBK1

In vitro pull-down assay using recombinant GST, GST-BACH2 381-481 or His-TBK1 in the presence or absence of 5 or 10 μM heme. There are four His residues in BACH2 381-481 that are capable of six-coordinate heme bonds. GST and GST-BACH2 381-481 were revealed with anti-GST antibody. TBK1 was revealed with anti-TBK1 antibody.

SUPPLEMENTAL FIGURES

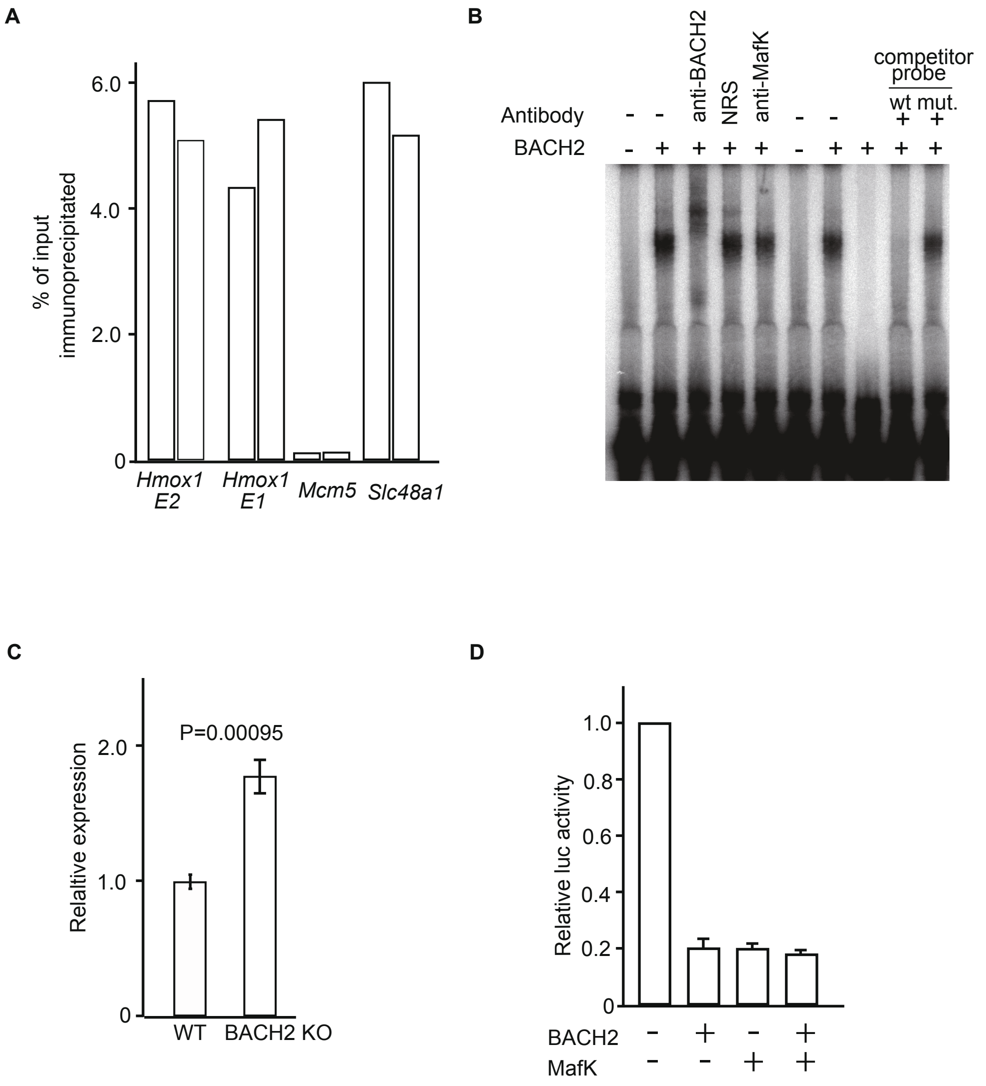

Figure S2, related to Figure 2

Figure S2. BACH2 directly represses *Slc48a1* gene

(A) Binding of BACH2 to the *Slc48a1, Hmox1 E1, E2 and Mcm5* loci was demonstrated by ChIP-qPCR in the BAL17 cell. **(B)** EMSA in the presence (+) or absence (–) of recombinant BACH2 (above lanes) plus oligonucleotide probes (Oligo) containing the MARE-like sequences (+) or mutated sequences (Mut.) of *Slc48a1*, or competitor oligonucleotides with wild-type (WT competitor) or mutated (Comp competitor)) sequence, incubated without antibody or with antibody to BACH2 (anti-BACH2-N2), normal rabbit serum (NRS), or antiserum to MAFK (anti-MAFK). **(C)** RT-qPCR analysis of *Slc48a1* mRNA expression in wild-type and BACH2 KO mouse splentic B cell. **(D)** HEK293T cells were transiently transfected with indicated reporter and effector plasmids. HEK293T cells were transiently transfected with indicated reporter and effector plasmids. The amounts of plasmids were as follows: pGL4-4.28-*Slc48a1*MARE luciferase reporter (2.0 μg), pEF seapansy (10 ng), pCMV BACH2 (200 ng) and pEF MAFK (200 ng).

SUPPLEMENTAL FIGURES

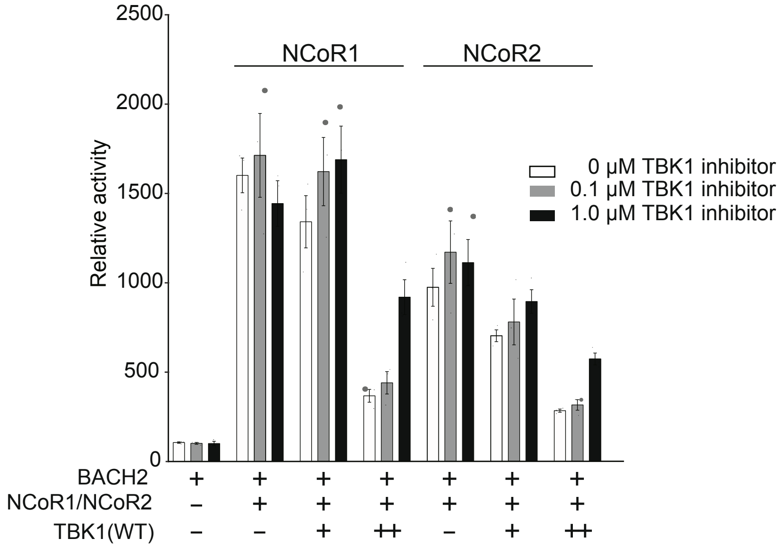

Figure S3, related to Figure 3

Figure S3. TBK1 regulates the interaction between BACH2 and NcoR1/2 transcriptional repressor complexes in a phosphorylation activity-dependent manner

Mammalian two-hybrid (M2H) assay with HEK293T cells in the presence or absence of the TBK1 inhibitor (MRT67307) from 0 to 1.0 μM concentration. BACH2, TBK1 (wild-type: WT) and co-repressor NCoRI or NcoR2 plasmids were transfected into HEK293T cells. After that, each sample was used for M2H assay.

SUPPLEMENTAL FIGURES

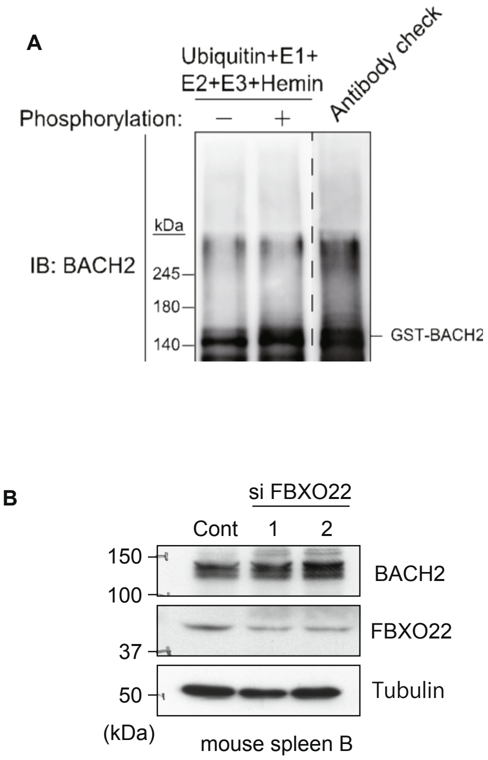

Figure S4, related to Figure 4

Figure S4. Increase in BACH2 protein levels by FBXO22 knockdown

(**A**) The Western blotting analysis indicating that the SCF^{FBXO22} E3 ligase complex not promote polyubiquitination of TBK1-mediated phosphorylation in BACH2 in the presence of 5 μM heme.

(**B**) Immunoblot analysis of BACH2 and FBXO22 in FBXO22 knockdown LPS-stimulated splenic B cells. Whole cell extracts from splenic B cells were prepared with RIPA Buffer. Immunoblot analyses were carried out with the indicated antibodies.

SUPPLEMENTAL FIGURES

```
                   342   347     352    357
331- SRSVSSPSCL RSLFGITKGV ESTGLPSTSQ QPLVRSSACP
                 382                            402
     FNKGISQGDL KTDYTPLAGN YGQPHVGQKD VSNFAMGSPL
     RGPGPETLCK QEGELDRRSV IFSASACDQP NTPVHSYSAV
                    464          472
     SNLDKDLSEP VPKSLWVGAG QSLPSSQAYS HSGLMADHLP
     GRIRPNTSCP VPIKVCPRSP PLETRTRTSS -520
```

HR-5 (Heme-regulated-5 sites) S352A,S357A,T382A,S402A,S472A

NHR-5 (Non-Heme-regulated-5 sites) S342A,T347A,S352A,S464A,S472A

ALL (8 sites) S342A, T347A, S352A,S357A,T382A,S402A, S464A, S472A

Figure S5, related to Figure 6

Figure S5. Phosphorylation site of BACH2IDR by TBK1

Phosphorylation site of BACH2IDR by TBK1, which was altered in the presence and absence of heme. Blue characters are sites that are more likely to be phosphorylated by TBK1 in the absence of heme. Red characters are sites that are more likely to be phosphorylated by TBK1 in the presence of heme.

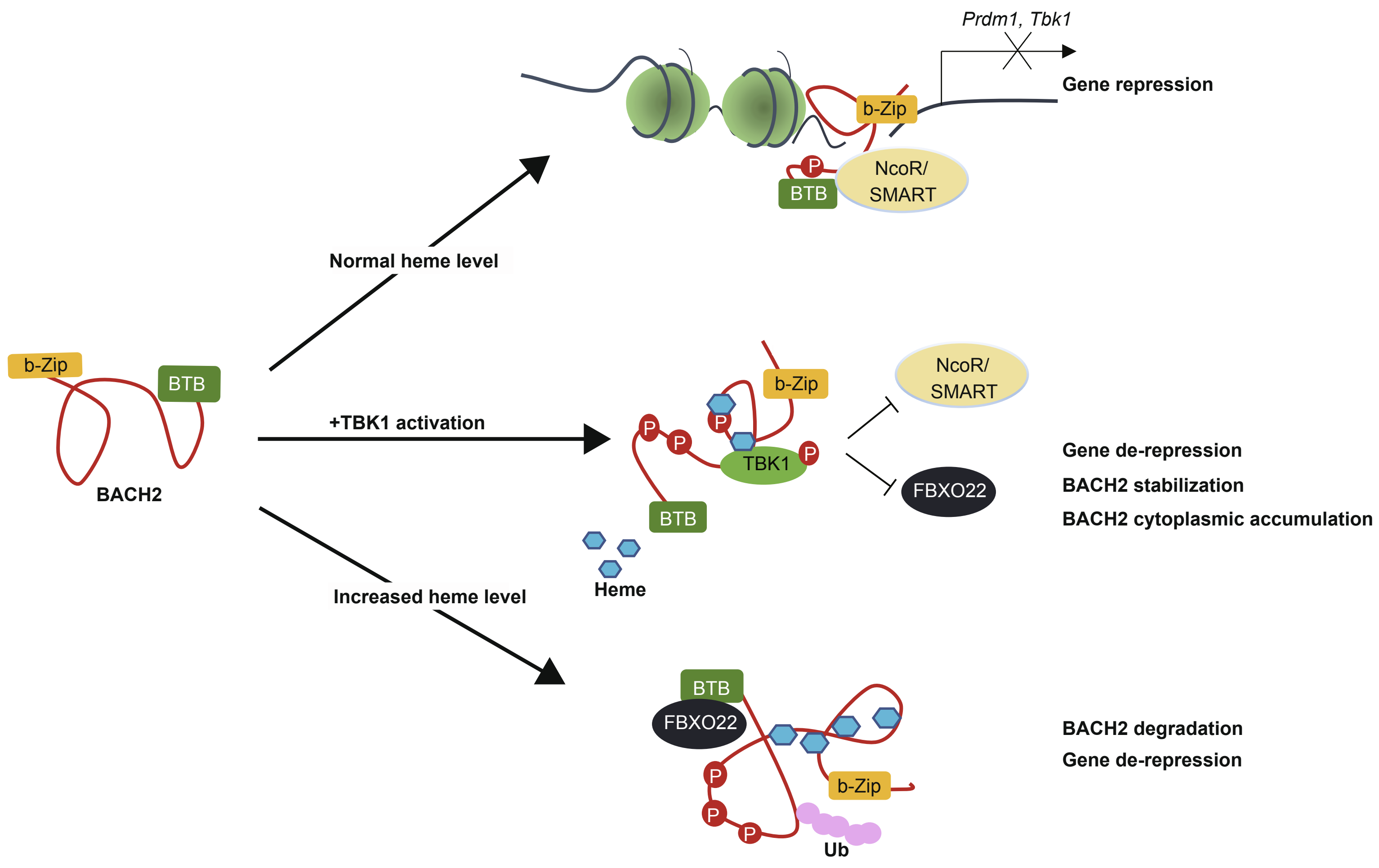

Figure S6, related to Discussion

Figure S6. Model of functional modulation of BACH2 by heme-signal

A model for the mechanism of the heme-mediated regulation of BACH2. Factors such as external stimulations gradually shift intracellular heme levels. The changes in heme concentrations induce a continuous structural change of BACH2, leading to loss of its transcriptional repression activity as well as changes in its protein-protein interactions. For details, see Discussion.
